# Effects of removing in-feed antibiotics and zinc oxide on the taxonomy and functionality of the microbiota in post weaning pigs

**DOI:** 10.1186/s42523-024-00306-7

**Published:** 2024-04-16

**Authors:** Juan M. Ortiz Sanjuán, Héctor Argüello, Raúl Cabrera-Rubio, Fiona Crispie, Paul D. Cotter, Juan J. Garrido, Daniel Ekhlas, Catherine M. Burgess, Edgar G. Manzanilla

**Affiliations:** 1 Pig Development Department, Teagasc Grassland Research and Innovation Centre, Moorepark, Fermoy, Co. Cork, Ireland; 2 Grupo de Genómica y Mejora Animal, Departamento de Genética, Facultad de Veterinaria, Universidad de Córdoba, Córdoba, Spain; 3 Departamento de Sanidad Animal, Facultad de Veterinaria, Universidad de León, León, Spain; 4 Teagasc Food Research Centre, Moorepark, Fermoy, Co. Cork, Ireland; 5 APC Microbiome Institute, University College Cork, Co. Cork, Ireland; 6 VistaMilk SFI Research Centre, Fermoy, Co. Cork, Ireland; 7 Department of Food Safety, Teagasc Food Research Centre, Ashtown, Dublin, Ireland; 8 School of Veterinary Medicine, University College Dublin, Dublin, Ireland

**Keywords:** Antimicrobial use, Diarrhea, Piglet, Shotgun sequencing, Swine

## Abstract

**Background:**

Post weaning diarrhoea (PWD) causes piglet morbidity and mortality at weaning and is a major driver for antimicrobial use worldwide. New regulations in the EU limit the use of in-feed antibiotics (Ab) and therapeutic zinc oxide (ZnO) to prevent PWD. New approaches to control PWD are needed, and understanding the role of the microbiota in this context is key. In this study, shotgun metagenome sequencing was used to describe the taxonomic and functional evolution of the faecal microbiota of the piglet during the first two weeks post weaning within three experimental groups, Ab, ZnO and no medication, on commercial farms using antimicrobials regularly in the post weaning period.

**Results:**

Diversity was affected by day post weaning (dpw), treatment used and diarrhoea but not by the farm. Microbiota composition evolved towards the dominance of groups of species such as *Prevotella* spp. at day 14dpw. ZnO inhibited *E. coli* overgrowth, promoted higher abundance of the family *Bacteroidaceae* and decreased *Megasphaera* spp. Animals treated with Ab exhibited inconsistent taxonomic changes across time points, with an overall increase of *Limosilactobacillus reuteri* and *Megasphaera elsdenii*. Samples from non-medicated pigs showed virulence-related functions at 7dpw, and specific ETEC-related virulence factors were detected in all samples presenting diarrhoea. Differential microbiota functions of pigs treated with ZnO were related to sulphur and DNA metabolism, as well as mechanisms of antimicrobial and heavy metal resistance, whereas Ab treated animals exhibited functions related to antimicrobial resistance and virulence.

**Conclusion:**

Ab and particularly ZnO maintained a stable microbiota composition and functionality during the two weeks post weaning, by limiting *E. coli* overgrowth, and ultimately preventing microbiota dysbiosis. Future approaches to support piglet health should be able to reproduce this stable gut microbiota transition during the post weaning period, in order to maintain optimal gut physiological and productive conditions.

**Supplementary Information:**

The online version contains supplementary material available at 10.1186/s42523-024-00306-7.

## Background

In the last decade, studies using high throughput sequencing methods have revolutionised knowledge about microbial communities, particularly in the gut [1]. The involvement of the microbiota in inflammatory and metabolic diseases, cancer, neurologic disorders and even behaviour are just a few examples of the recent discoveries and impacts on host health from this microbial community [1, 2].

This research topic is also attracting the interest of animal researchers, particularly in livestock, and different studies have detailed the microbiota composition in different species [3–5]. For instance, numerous studies describe in detail the evolution of the pig microbiota, at genus [5, 6] and species [7] taxonomic levels, along their production cycle. These and other studies describe the sudden disruption of the microbiota in piglets weaned under intensive commercial condition [8, 9]. Weaning on intensive farms usually takes place between the third and the fifth week of age. Piglets are separated from the sow, moved into new pens, mixed with other litters and fed a solid diet. This abrupt change including environmental, social and nutritional factors severely impacts the composition of the microbiota. Indeed, the microbiota dysbiosis which occurs at weaning is one of the major factors prompting post weaning diarrhoea (PWD), the most frequent health problem on commercial pig farms and a major reason for antimicrobial use [10]. PWD is usually associated with *Escherichia coli*, enterotoxigenic *E. coli* (ETEC) strains mostly, but also other pathogens such as enteroadherent strains of *E. coli*, *Salmonella enterica*, *Lawsonia intracellularis* or Rotavirus.

For more than five decades, control of PWD was addressed by the systematic use of antibiotics and heavy metals, particularly, zinc oxide [8, 10–12]. These compounds have an antimicrobial effect on targeted pathogens (e.g., ETEC), but also their antimicrobial effect may go beyond the target pathogen and impact the broader microbiota of piglets at weaning [13–16], thereby shaping the microbiota composition, when used [17]. This selection towards microorganisms which adapt better to their presence may exert a competitive exclusion effect against ETEC at weaning. Current knowledge on the microbiota changes caused by antibiotics and therapeutic ZnO are constrained by the disparity of results. The observed variability is linked to external factors influencing these studies, such as design, methodology used or management factors. As a consequence, to date, it has been difficult to identify common changes in the microbiota across farms or even studies.

In this scenario, the present study was designed to compare the effects of in-feed antibiotics (Ab) and therapeutic doses of zinc oxide (Zn) with non-medicated animals (Ct) on the development of the post-weaning microbiota on four commercial pig farms. The design of the study and data analyses accounted for the variability among the pig farms. This design overcomes individual farm variability and idenfity common microbial features shaped by post weaning treatments, providing interesting results which should be considered in ZnO and antibiotic replacement.

## Materials and methods

### Experimental design

In this study, we assessed the effect of three different dietary treatments, antibiotic (Ab), therapeutic zinc oxide (Zn) or non-medicated control (Ct), on four farms where antibiotics and Zn were used regularly at post weaning period. Prior to this study the four farms had tried to withdraw Ab and Zn treatments from the feed but it resulted in diarrhoea outbreaks and negatively impacted performance figures. Farms A, B and C used the control diet + 250 mg/Kg of sulphadiazine and 50 mg/Kg of trimethoprim as Sulfoprim 15% (Univet Limited, Ireland), while on farm D the control diet was supplemented with 400 mg/Kg of amoxicillin as Stabox 5% (Virbac, France). The Zn group was fed with the control diet + 2500 mg/Kg of zinc oxide as Pigzin (DSM Animal nutrition, United Kingdom). The dietary treatments were administered in the first two weeks post weaning and samplings were scheduled at days 0, 7 and 14 post weaning (0dpw, 7dpw, and 14dpw. respectively). The same experiment was replicated twice on each of the four farms (A, B, C, D). Piglets were weaned at 4 weeks of age and moved to the weaning rooms, where the groups were balanced by weight. Pigs were fed a dry pelleted starter diet fulfilling nutritional requirements (NRC, 2012). Feed was provided manually in bags and intake was recorded at pen level by counting/weighing the bags weekly.

### Sample collection, DNA extraction and library preparation

At sampling days 0, 7 and 14dpw, three random freshly voided faecal samples from three pigs per treatment pen were collected and pooled into a 100mL cup where they were mixed and homogenised using a sterile 210 × 11 mm sampling spatula, and then transferred to 1.5mL microcentrifuge tube using a sterile 140 × 7 mm conical steel spatula. On day 7dpw, diarrhoea samples were collected from any pen included in the trial where fresh diarrhoea was observed on pen walls, avoiding the part of faeces in direct contact with the surface, using the same procedure described above. Samples were immediately snap-frozen on dry-ice upon collection and transported to the research facilities where they were stored at -80° C until processing. The DNA from the faecal samples was extracted using the QIAamp PowerFecal Pro DNA Kit (Qiagen, Crawley, West Sussex, UK) following the manufacturer’s instructions, using 200 ± 50 mg of faecal content. A Qubit fluorimeter (Qubit 3, Invitrogen) was used to determine the total DNA concentration. Paired-end sequencing libraries were prepared from the extracted DNA using the Illumina Nextera XT Library Preparation Kit (Illumina Inc., San Diego, CA) followed by sequencing on the Illumina NextSeq 500 platform using high-output chemistry (2 × 150 bp) according to the manufacturer’s instructions. The library size from each sample was assessed on an Agilent Technology 21,000 Bioanalyzer using a High Sensitivity DNA chip.

### Bioinformatic analysis

Raw reads were filtered using trimmomatic v0.38 [18]. An average quality threshold score of 25 in a sliding window of 10 base pairs was used to trim reads below the threshold. A minimum length of 150 base pairs was ensured for all reads. Bowtie2 v2.4.4 [19] was used to map the reads against host and human reference genomes, keeping the unmapped reads for the downstream analysis. Reference genomes were downloaded from Bowtie2 website. Read duplicates were removed using a bbmap 38.22 tool called clumpify.sh [20]. Taxonomic assignment of the sequences was carried out using Kaiju v1.7.4 [21]. Functional profiles were assigned using SUPER-FOCUS v0.0.0 [22]. SUPER-FOCUS software collapses the functions assigned to a metagenome in three levels: from level 1 being the broadest categories (SF1), to level 3 being the most specific functions (SF3). Abundance of Pfam protein families and domains was obtained from UniRef90 gene families using HUMAnN v3.0 [23]. Pfam is a comprehensive Database of Protein Domain Families [24]. Processed reads were assembled into contigs using metaSPAdes pipeline from SPAdes v3.15.3 [25]. Mass screening of assembled contigs for *E. coli* virulence factors was performed with ABRicate (v1.0.1; https://github.com/tseemann/abricate/), using the Ecoli_VF database (https://github.com/phac-nml/ecoli_vf).

### Statistical analysis

Analyses were carried out in R v4.2.1 [26], studying differences on microbiota composition and functionality amongst the variables day post weaning (0, 7 or 14dpw), treatment (Ct, Ab or Zn), farm (A, B, C, D) and faecal consistency (normal or diarrhoea). In-feed dietary treatment effects on the microbiota were studied globally and within each faecal consistency and dpw level. For first ordination and clustering, and differential abundance analysis within each faecal consistency and dpw, faecal consistency and dpw variables were merged into a new variable called “Consistency-dpw”: Faecal_0dpw, Faecal_7dpw, Diarrhoea_7dpw, and Faecal_14dpw. To ensure a balanced statistical arrangement, diarrhoea samples were not included as part of the global analysis, nor when studying differences among sampling time points. Alpha and beta diversities were both computed at the species and functional level using the phyloseq R package v1.40.0 and vegan R package v2.6-2, respectively [27, 28]. The estimation of alpha diversity in taxonomic and functional data, included an initial analysis of alpha diversity indexes computed using raw reads. Potential differences in diversity between sequencing runs associated to variations in sequencing depth was checked using Kruskal Wallis test or ANOVA depending on their data distribution. When significant differences were found for any of the computed indexes, data was normalized using rarefaction. Raw reads classified by kaiju were normalized using rarefaction by the minimum total number of sequences using the function rarefy_even_depth from the phyloseq R package. Samples that did not reach the richness plateau in the rarefaction curve were removed from the analysis of alpha diversity. Alpha diversity was estimated by Species richness, Chao1, Simpson, and Shannon and Pielou evenness indexes. Statistical differences in alpha diversity indexes were tested, after testing their distribution (Shapiro Wilk test, *P* < 0.05), with ANOVA followed by pairwise comparison with Tukey (car v3.0.10) [29], multcompView v0.1.8 [30] and lsmeans v2.30.0 [31] R packages) or by Kruskal-Wallis followed by pairwise Wilcoxon test (stats v4.0.2 R package) [26], when data did not follow a normal distribution. Wilcox.exact from exactRankTests v0.8-35 R package function was used for exact p-value estimation of data with ties [32]. Beta diversity and ordination of samples were performed in relative abundance transformed data, by Non-Metric Multidimensional Scaling (NMDS) of previously calculated Bray Curtis distances between samples of species and functional abundance data, using the Vegan package in R. Ordination of virulence factors data was performed by NMDS of distances calculated using Simple matching coefficient [33]. Distance between groups centroids was tested with PERMANOVA (adonis2 and pairwise adonis) [34]. Factors and species influencing the ordination were assessed by linear models fitting on the ordination results (envfit function in Vegan R package). All p-values were adjusted by Benjamini-Hochberj (BH) approach. For fitting species in ordination space, taxa and pathways were filtered, keeping the top 15 species and functions with the highest mean abundance across samples. Taxa and function abundance analyses among treatments analysed globally and within each consistency-dpw level, were performed using Linear Discriminant Analysis Effect Size (LEfSe [35]). The treatment factor was used as class, selecting an alpha cut-off of 0.05 and a LDA threshold of 2. Species and functions explaining differences between treatments were determined by LEfSE using Kruskal-Wallis test (*P* < 0.05) followed by linear discriminant analysis. Analysis of virulence factors (presence/absence) among treatments in each consistency-dpw level was performed using pairwise_fisher_test from rstatix v0.7.1 package [36]. Figures were produced with R and subsequently arranged using inkscape software v1.0.2 [37].

## Results

### Microbial diversity and richness are defined mostly by day post weaning but are also affected by treatment and diarrhoea occurrence

**Fig. 1:**
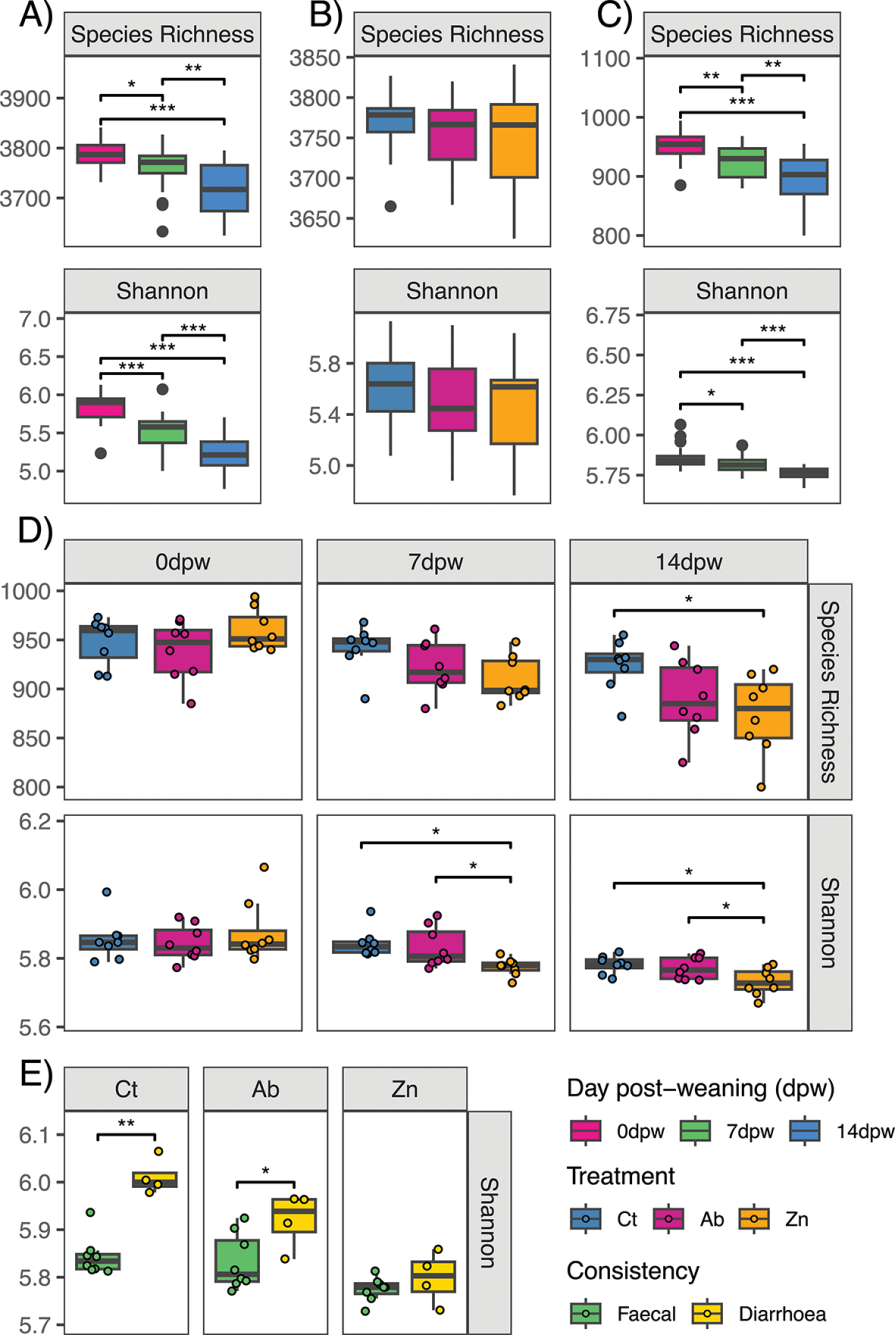
Analysis of microbiota α-diversity at species and functional levels, by day post weaning (dpw) and treatment. (**A**) Results of α-diversity at species level, by day post weaning. (**B**) Alpha diversity values by treatment factor, at species level. (**C**) Results of α-diversity, at functional level (Super-focus level 3 category), by dpw. (**D**) Results of α-diversity analysis by treatment betwen each day post weaning, performed at functional level (Super-focus level 3 category). (**E**) Results of α-diversity analysis in 7dpw samples, comparing normal and diarrhoeic faeces and performed at functional level (Super-focus level 3 category) in each dietary treatment group. *P < 0.05, **P < 0.01, and ***P < 0.001. Sequences were taxonomically and functionally assigned using Kaiju and Super-focus, respectively

Alpha diversity analysis was performed estimating richness and evenness indexes (Species richness, Chao1, Simpson, Shannon, Pielou’s evenness). Differences among treatments and over dpw were analysed for all the computed indexes either at species or functional level (Fig. 1 and Supplementary figure S1). Diversity decreased over the post weaning period for all indexes at species (Fig. 1A) and functional level (Fig. 1C). The treatment did not affect diversity at species level (Fig. 1B) but it affected functional diversity on days 7 and 14 (Fig. 1D), when animals fed with the ZnO diet showed lower diversity for some of the indexes evaluated. As expected, no differences were found at 0dpw between treatments. At 7dpw, the Ct and Ab groups exhibited higher Shannon and Simpson diversity values than the Zn group (Fig. 1D and Supplementary figure S1D, respectively) and also the Ct group had greater Pielou evenness than the Zn group (Supplementary Fig. S1SD). At 14dpw, the Ct group had greater Species richness and Simpson diversity values than the Zn group (Fig. 1D and Supplementary figure S1D, respectively), and the Ct and Ab groups also exhibited higher values than the Zn group in Shannon index (Fig. 1D). Finally, we compared the alpha diversity in normal and diarrhoea faecal samples collected at 7dpw. At species level, diarrhoeic faeces showed lower diversity for Chao1 and Simpson indexes only in the Ct group (Supplementary figure S1E). At a functional level, diarrhoeic samples in the Ct group exhibited higher diversity in the indexes of Shannon, Pielou evenness and Simpson, compared to non-diarrhoeic pigs (Shannon: Fig. 1E; Pielou evenness and Simpson: Supplementary figure S1F). Diarrhoeic samples collected from animals in the Ab medicated-feed group also showed higher diversity than non-diarrhoeic animals, but only for Shannon and Simpson indexes, and these differences were smaller (Fig. 1E and Supplementary figure S1F).

**Fig. 2:**
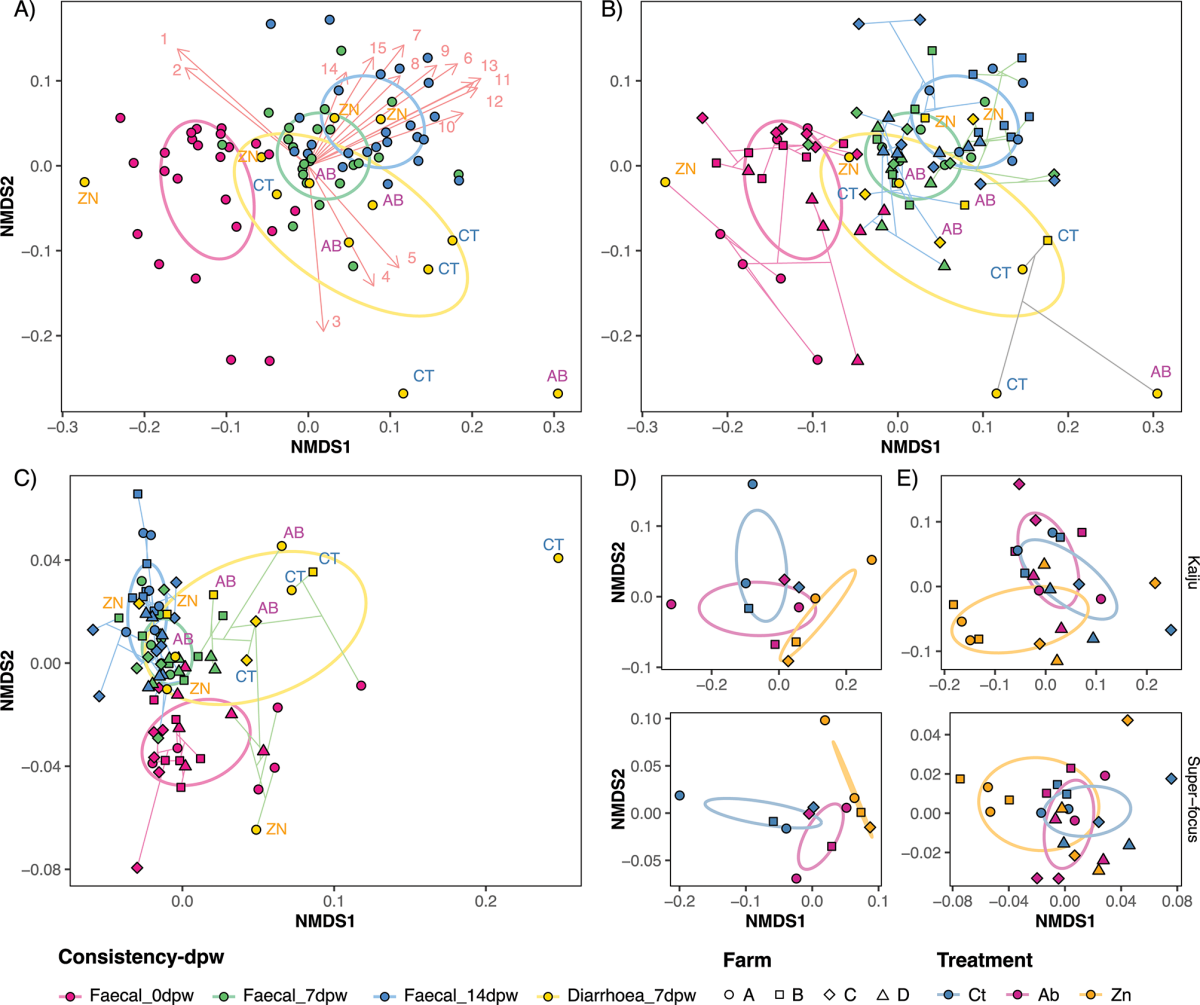
Analysis of microbiota β-diversity by type of sample (consistency), day post weaning and treatment. (**A**-**C**) Samples ordination using Bray-Curtis distances at species (**A**-**B**) and functional level (C) in a NMDS; coloured according to faecal consistency and dpw factor (Consistency-dpw). (**A**) Arrows display the top 15 species with the highest mean abundance returned by “envfit” model, influencing the ordination of samples (Arrows showing BH p.adjusted significant species; Arrows length shows the strength of each species influencing the ordination of samples). Species fitted onto ordination are indicated as numbers, ordered according to its NMDS coordinates. 1: *Oscillibacter* sp. PEA192, 2: *Clostridiales* bacterium CCNA10, 3: *Escherichia coli*, 4: *Megasphaera elsdenii*, 5: *Limosilactobacillus reuteri*, 6: *Anaerobutyricum hallii*, 7: *Faecalibacterium prausnitzii*, 8: *Roseburia hominis*, 9: *Prevotella ruminicola*, 10: *Eubacterium rectale*, 11: *Prevotella dentalis*, 12: *Hoylesella enoeca*, 13: *Prevotella oris*, 14: *Blautia pseudococcoides*, 15: *Lachnospiraceae* bacterium GAM79. (**B**) Ward Clustering of Bray Curtis distances ploted onto a NMDS. The dendrogram was split into four branches and coloured according to the samples’ arrangement within the NMDS space. (**C**) Ordination of samples using Bray-Curtis distances at functional level of Super-focus level 3 category. (**D**) Ordination of 7dpw diarrhoea samples, using Bray-Curtis distances, onto a NMDS, coloured by treatment both at species (top) and functional (bottom) levels. (**E**) Ordination of 14dpw faecal samples, using Bray-Curtis distances, onto a NMDS, coloured by treatment both at species (top) and functional (bottom) levels. Ellipses represent covariance for each factor level: faecal consistency and dpw in figures **A**-**C**; and treatment in figures **D**-**E**. Treatments to which each sample belonged to are indicated for diarrhoea samples in figures **A**-**C** as CT(Ct), AB (Ab) and ZN (Zn). Farm factor is indicated by the shape of the points in figures **B**-**E**, coloured according to faecal consistency and dpw (**B**-**C**) or treatment (**D**-**E**)

Ordination of samples by Bray-Curtis distance into a non-metric multidimensional scaling (NMDS) revealed differences in microbiota composition by treatment, day post weaning, as well as by farm and faecal consistency factors, either at species or functional level (Fig. 2A and B at Species level, Fig. 2C at functional, and Supplementary tables S1 and S2 for both levels). The analysis including treatment and dpw (Supplementary table S1) revealed influences in the ordination of both species and functional datasets. Pairwise level comparison confirmed differences in microbiota composition among dpw but not for treatments (Supplementary table S2). Despite the lack of an interaction and based on the NMDS plots, further analyses were carried out for the treatment within each sampling time point (Supplementary table S3, Fig. 2D and E). In this analysis, the Zn group showed changes in the taxonomic analysis at 14dpw compared to the Ab and Ct groups (Supplementary table S3, Fig. 2E). The analysis of diarrhoeic vs. normal faeces at 7dpw revealed significant differences in the ordination of taxonomic and functional traits, but further analyses did not show significant differences among treatment groups (Fig. 2D; Supplementary table S4). Finally, the farm showed no effect on the taxonomic or functional analysis.

### Weaning and diarrhoea occurrence define major taxa clusters

**Fig. 3:**
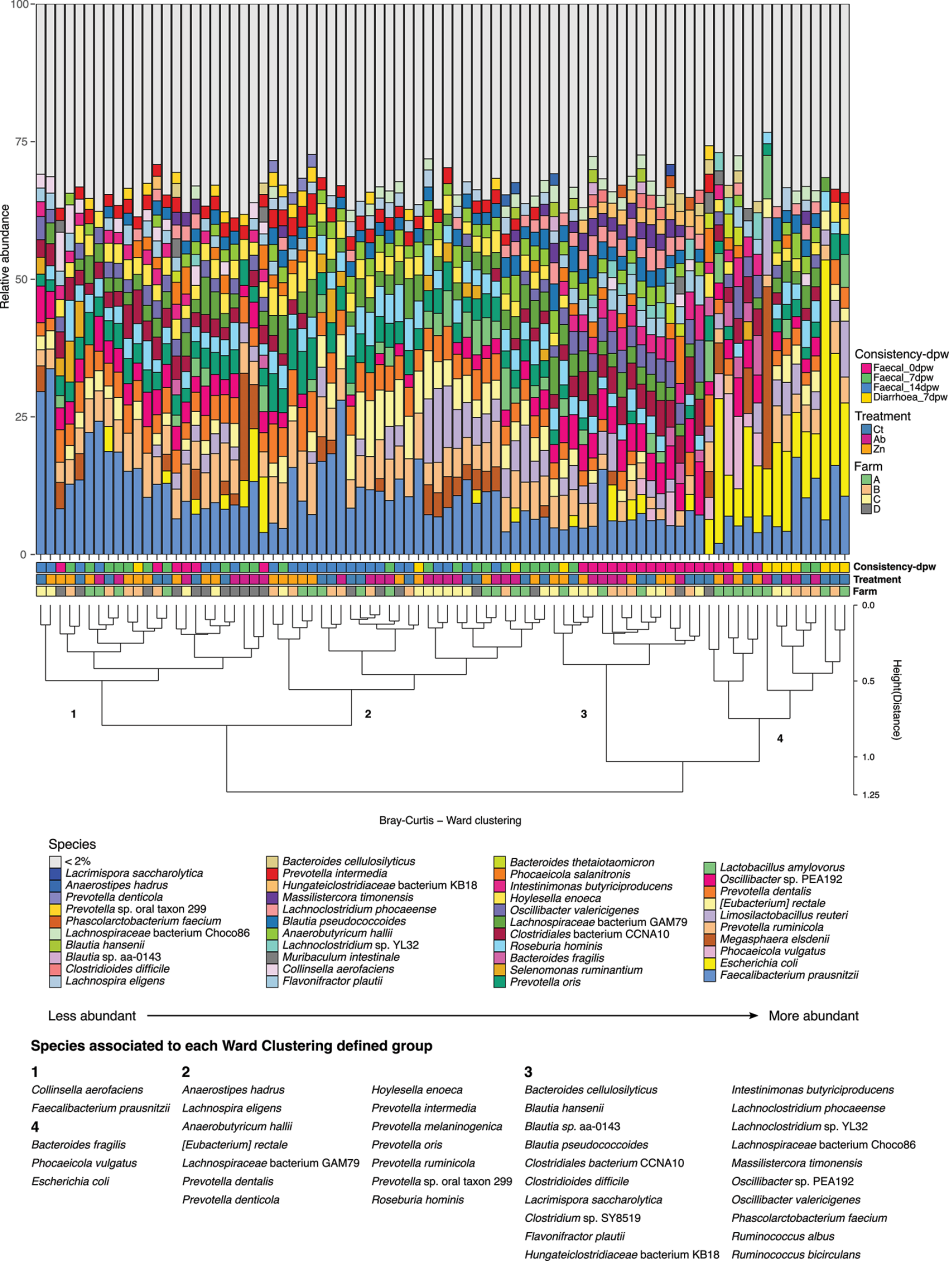
Relative abundance of the main species in each sample. The profiles of samples are ordered by Ward clustering of the squared Bray-Curtis distances between samples. The cluster dendrogram represents the similarity between samples regarding its microbial composition. Variables information for each sample (from lower to upper level: Farm, Treatment, Consistency-dpw) are indicated in the coloured squares below the bars. Numbers besides each branch refers to the main groups defined by Ward clustering, for which indicator species of each group determined by ‘*multipatt*’ are shown at the bottom part of the figure. Taxonomic identification of sequences was performed using Kaiju

Further analysis of sample clusters and representative species across samples was performed by Ward clustering of Bray-Curtis distances, which arranged the samples into four major clusters (Fig. 3). Cluster 1 included samples from all time points and treatments. Cluster 2 included only samples from 7dpw and 14dpw. Cluster 3 included almost exclusively 0dpw samples (13 out of 16 samples). Cluster 4 grouped most of the Diarrhoea_7dpw samples (8), two samples from 7dpw and five from 0dpw time points. The species associated with each cluster are also shown in Fig. 3. Samples in cluster 1 were linked with *Collinsella aerofaciens* and *Faecalibacterium prausnitzii*. Cluster 2 included species of *Prevotella* spp. and order *Eubacteriales (Anaerobutyricum hallii*, *Anaerostipes hadrus, Lachnospiraceae* bacterium GAM79, *Lachnospira eligens* and *Roseburia hominis)*. Patterns in cluster 3 were defined by several species of *Ruminococcus* spp., *Blautia* spp., *Oscillibacter* spp., *Lachnoclostridium* spp., *Bacteroides cellulosilyticus, Clostridiales* bacterium CCNA10, *Clostridioides difficile, Lacrimispora saccharolytica, Clostridium* sp. SY8519, *Intestinimonas butyriciproducens, Flavonifractor plautii, Hungateiclostridiaceae* bacterium KB18, *Lachnospiraceae* bacterium Choco86, *Massilistercora timonensis* and *Phascolarctobacterium faecium*. Cluster 4 was associated with *Bacteroides fragilis* and *Phocaeicola vulgatus* and *Escherichia coli*.

### Microbial composition evolves rapidly during the post weaning period and is affected by treatments and diarrhoea occurrence

**Fig. 4:**
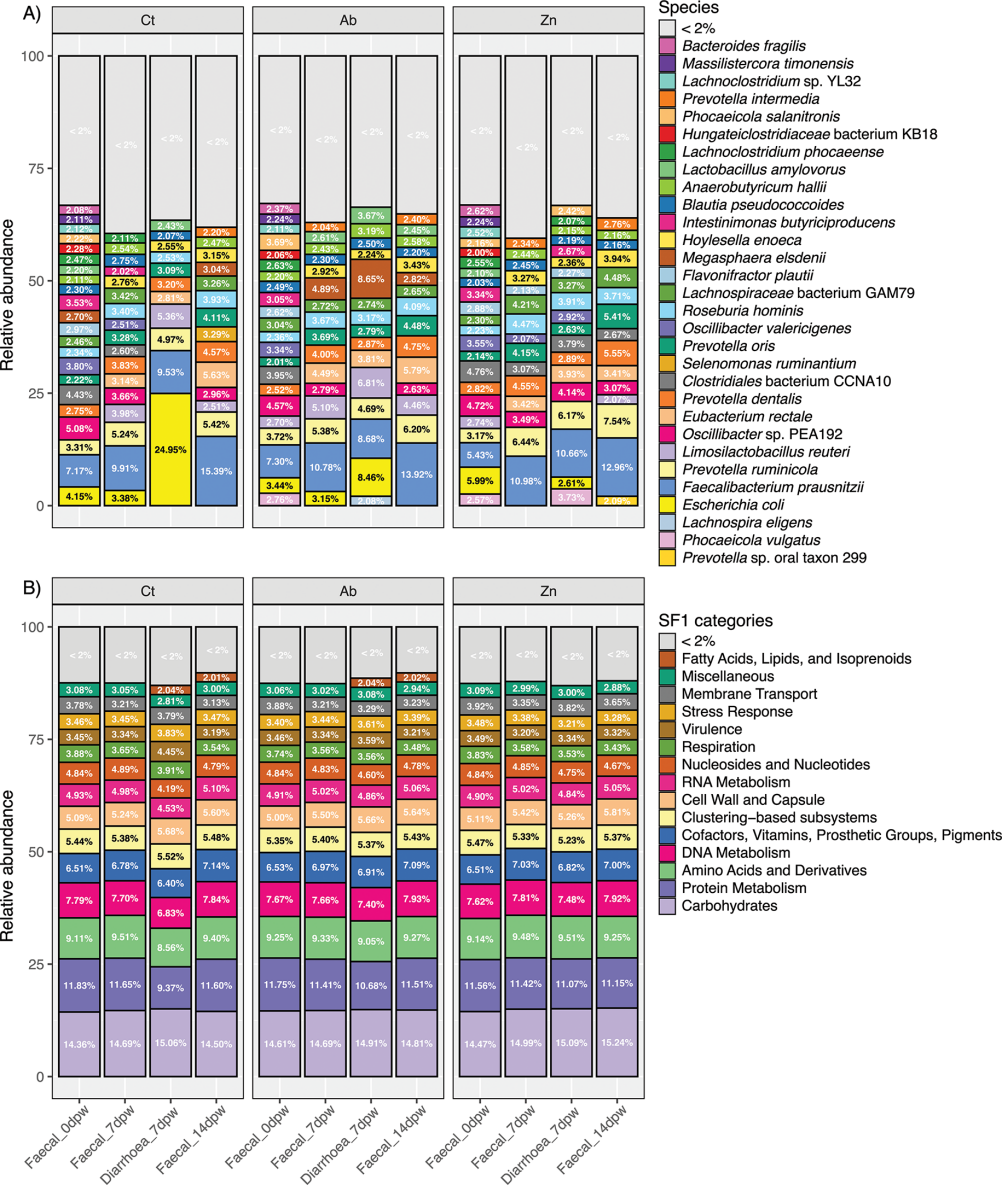
Faecal microbiota composition of weaning pigs at days 0, 7 and 14 post weaning (dpw). (**A**). Mean relative abundance of the most representative species in each consistency-dpw group. (**B**) Mean relative abundance of the most representative functional categories of super-focus level 1 in each consistency-dpw group. Consistency-dpw groups are separated by dietary treatment. Taxonomic and functional profiling of sequences was performed using Kaiju and Super-focus, respectively

Next, to compare the microbiota evolution throughout the two weeks post weaning in each treatment, the mean relative abundance species profiles of each consistency-dpw level were investigated separately (Fig. 4A). In non-diarrhoeic samples, *F. prausnitzii* was the dominant species regardless of the dpw factor. Samples from day 0dpw revealed a notably similar pattern between treatments with, as seen in alpha diversity analyses, an even distribution of low-abundant species and a few dominant species such as *E. coli*, *F. prausnitzii*, *Oscillibacter* spp., and *Clostridiales* bacterium CCNA10. The relative abundance of some groups was affected after one week post weaning (Faecal_7dpw). Increased abundance was observed for species such as *Prevotella* spp., *Lachnospiraceae* bacterium GAM79 or *L. reuteri*; while decreased abundance was detected in *Intestinimonas butyriciproducens* and *Oscillibacter* spp. in the Ct group (Fig. 4A). Other species such as *Eubacterium rectale* or *Prevotella intermedia* were detected for first time in Ab and Zn treated groups. In addition, an increase in *Megasphaera elsdenii* abundance was observed in Ab treatment group at 7dpw and 14dpw. The relative abundance of *L. reuteri* and *L. amylovorus* was affected by Zn treatment, with lower abundance within the two weeks of study. A clear effect seen among treatments was the lower abundance or absence of *E. coli* in Ab and Zn groups in samples from 7dpw and 14dpw. In the 7dpw diarrhoea samples, *E. coli* reached a relative abundance of 24.95% in the Ct group samples while its relative abundance was lower in the diarrhoea samples of Ab and Zn treated groups (8.46 and 2.61%, respectively). Other bacteria affected by treatments in diarrhoea samples were *L.* bacterium GAM79, which was absent in Ct samples and maintained a similar relative abundance in Ab and Zn treatment groups. Species seen only in Zn diarrhoea samples were *C.* bacterium CCNA10 and *P. vulgatus* (3.80 and 3.70%, respectively). Finally, Faecal_14dpw microbiota was dominated by *Prevotella* spp. (*P. ruminicola, P. dentalis, H. enoeca* and *P. oris*), with the presence of other species at a lower relative abundance, such as *M. elsdenii*, observed in the Ct and Ab groups while absent in the Zn group, *Selenomonas ruminatum* was only found in Ct animals, and *C.* bacterium CCNA10 and *P.* oral taxon 299 in the Zn treated group (2.66 and 2.01%, respectively). Profiles of mean relative abundance of each level of consistency-dpw were compared amongst farm and treatments. These profiles showed a particular consistency in the inhibitory effect of ZnO in *E. coli* growth across the three farms where diarrhoea was observed. These profiles also became more similar among farms in samples collected at 14dpw. (Supplementary figure S2).

Functional data grouped in SF level 1 categories (Fig. 4B) revealed that the most abundant categories in all consistency-dpw samples reaching together approximately 50% were: (i) Carbohydrates, (ii) Protein Metabolism, (iii) Amino acids and derivatives, (iv) DNA metabolism, and (v) Cofactors, vitamins, prosthetic groups, and pigments. These functions reached less than 50% in Diarrhoea_7dpw samples from the Ct diet, in which the category Virulence accounted for 4.45%, compared to a range of 3.19 to 3.59% in the rest of the samples.

### Species differential abundance is defined by the binomial treatment-time

**Fig. 5:**
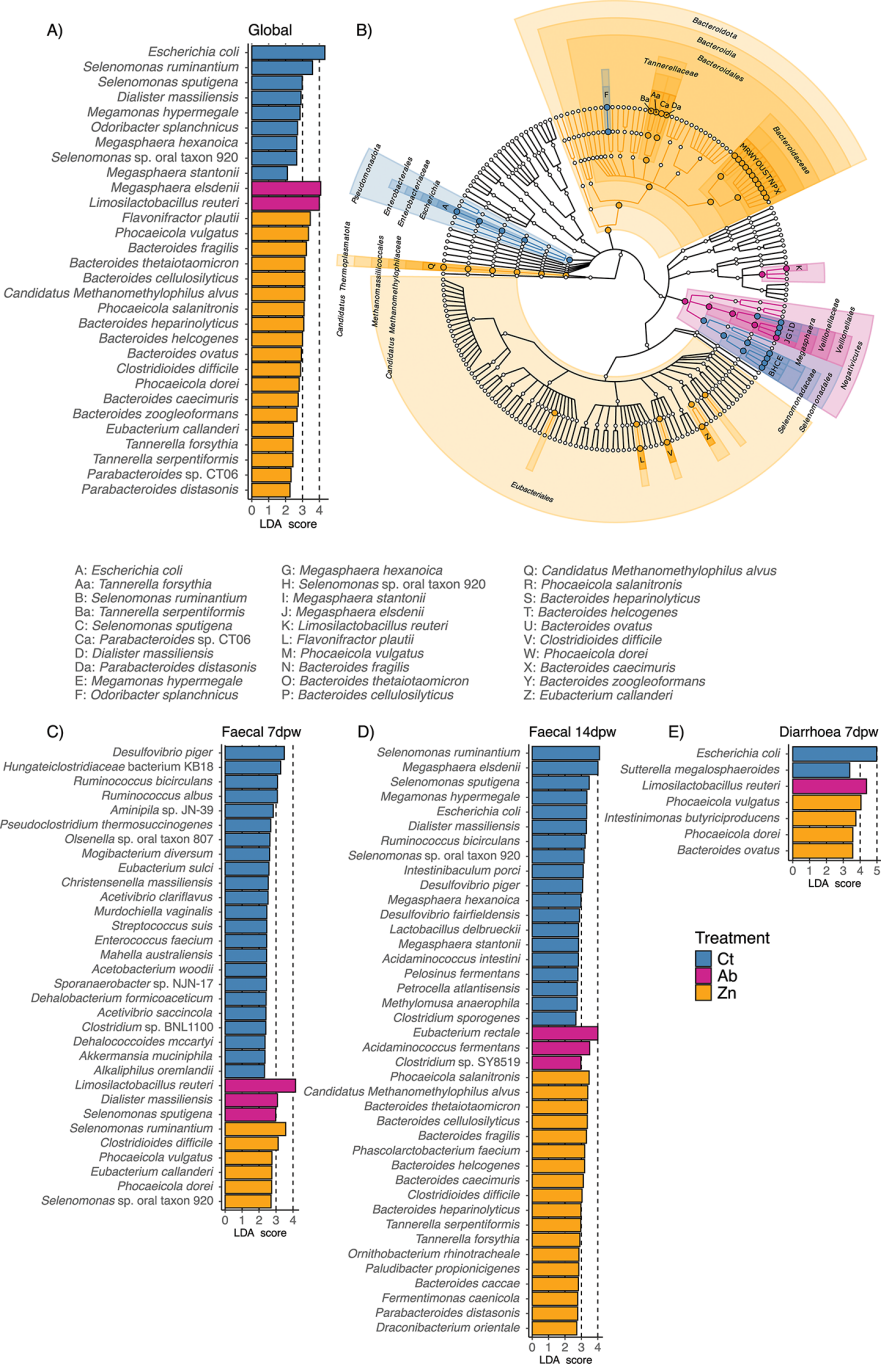
Differences in species abundance, returned by LEfSe, most likely explaining the differences among dietary treatments. (**A**) Species associated with each dietary treatment in the analysis of global species data. (**B**) Taxa associated with each dietary treatment in the analysis of global species data. Significant species are coloured according to the treatment to which they are associated with, and are annotated in the cladogram as letters, which can be identified below. (**C**) Species associated with each treatment at day 7 post weaning (7dpw). (**D**) Species associated with each treatment at day 14 post weaning (14dpw). (**E**) Species associated with each treatment, in diarrhoea samples of day 7 post weaning (7dpw). LEfSe: Linear discriminant analysis Effect Size

Analysis of the global dataset (Fig. 5A and B) and separated by faecal consistency and dpw factors (Fig. 5C, D and E) revealed different results in each subset. Global analysis linked several species within *Selenomonadaceae* (*Selenomonas* spp., *Megamonas hypermegale*), *Diallister massiliensis* and two species from *Megasphaera* spp. (*M. hexagonica* and *M. stantonii*) to animals fed the Ct diet. *M. elsdenii* abundance was higher in Ab treated animals, while Zn treated animals had higher relative abundance of *Bacteroides* spp., *Phocaeicola* spp., *Clostridioides difficile*, *Eubacterium callanderi*, *Flavonifractor plautii*, *Phascolarctobacterium faecium*, *Candidatus Methanomethylophilus alvus, Tannerella* spp. and *Parabacteroides* spp. (Fig. 5A).

Analyses by dpw revealed no differences among dietary treatments at 0dpw, except for the species *Phocaeicola salanitronis* which was enriched in the Ab group microbiota (Supplementary figure S3A). At day 7dpw (Fig. 5C, Supplementary figure S3B), faecal samples of the Ct group showed a higher abundance of *Desulfovibrio piger*, species associated with the family *Oscillospiraceae* (*Acetivibrio clariflavus* and *Acetivibrio saccincola*), *Ruminococcus* (*R. albus*, *R. bicirculans*), *Hungateiclostridiaceae* bacterium KB18, *Pseudoclostridium thermosuccinogenes*, *Clostridium* sp. BNL1100, *Alkaliphilus oremlandii*, *Akkermansia muciniphila*, *Dehalococcoides mccartyi*, *Olsenella* sp. oral taxon 807, *Murdochiella vaginallis*, *Enterococcus faecium* and *Streptococcus suis*, as well as *Christensenella massiliensis, Dehalobacterium formicoaceticum*, and several species within the family *Eubacteriales Family XIII Incertae Sedis* (*Aminipila* sp. JN-39, *Eubacterium sulci*, *Mogibacterium diversum*). Ab treated animals were associated with a higher abundance of *Selenomonas sputigena* and *D. massiliensis*. Finally, Zn treated animals exhibited higher relative abundance of *Selenomonas ruminantium, Selenomonas* sp. oral taxon 920, *Clostridioides difficile*, *Phocaeicola vulgatus, Phocaeicola dorei* and *Eubacterium callanderi*.

Faecal samples collected at 14dpw (Fig. 5D, Supplementary figure S4) revealed the association of *Megasphaera* spp. (*M. elsdenii*, *M. hexagonica*, *M. stantonii*), several species within the *Selenomonadaceae* (*S. ruminantium*, *S.* sp. oral taxon 920, *Selenomonas sputigena*, and *M. hypermegale*) and *Sporomusaceae* familiae *(Methylomusa anaerophila, Pelosinus fermentans)*, as well as *Diallister massiliensis, Acidaminococcus intestinii*, *Lactobacillus delbrueckii, E. coli*, *Desulfovibrio fairfieldensis* and *D. piger* to the Ct diet.

Samples from Ab treated animals were associated with *Acidaminococcus fermentans*, *E. rectale* and *Clostridium* sp. SY8519, whereas samples from Zn treated animals exhibited a higher abundance of species belonging to *Tanerellaceae* (*Tannerella forsythia*, *Tannerella serpentiformis*, *Parabacteroides* sp. CT06, *Parabacteroides distasonis*) and *Bacteroidaceae* families (*B. thetaiotaomicron*, *P. salanitronis*, *B. heparinolyticus*, *B. helcogenes*, *B. fragilis*, *B. cellulosilyticus*, *B. caecimuris* and *B. caccae*), as well as *Phascolarctobacterium faecium, Clostridioides difficile*, *Ornithobacterium rhinotracheale*, *Candidatus Methanomethylophilus alvus*, *Paludibacter propionicigenes, Fermentimonas caenicola* and *Draconibacterium orientale.*

Diarrhoea_7dpw samples (Fig. 5E, Supplementary figure S5) had a higher abundance of *E. coli* and *Suterella megalosphaeroides* in the Ct group, *Bacteroidaceae* family (*P. vulgatus*, *P. dorei* and *B. ovatus*) and *lntestinimonas butyriciproducens* in the Zn group, and *L. reuteri* in the Ab group.

The relative abundance of the species associated with each treatment in the analysis of each consistency-dpw level were compared across farms. Overall, most of the species included in the analyses exhibited a similar treatment-trend or relative abundance regardless of the farm (Supplementary figure S6).

### Functional differential abundance

**Fig. 6:**
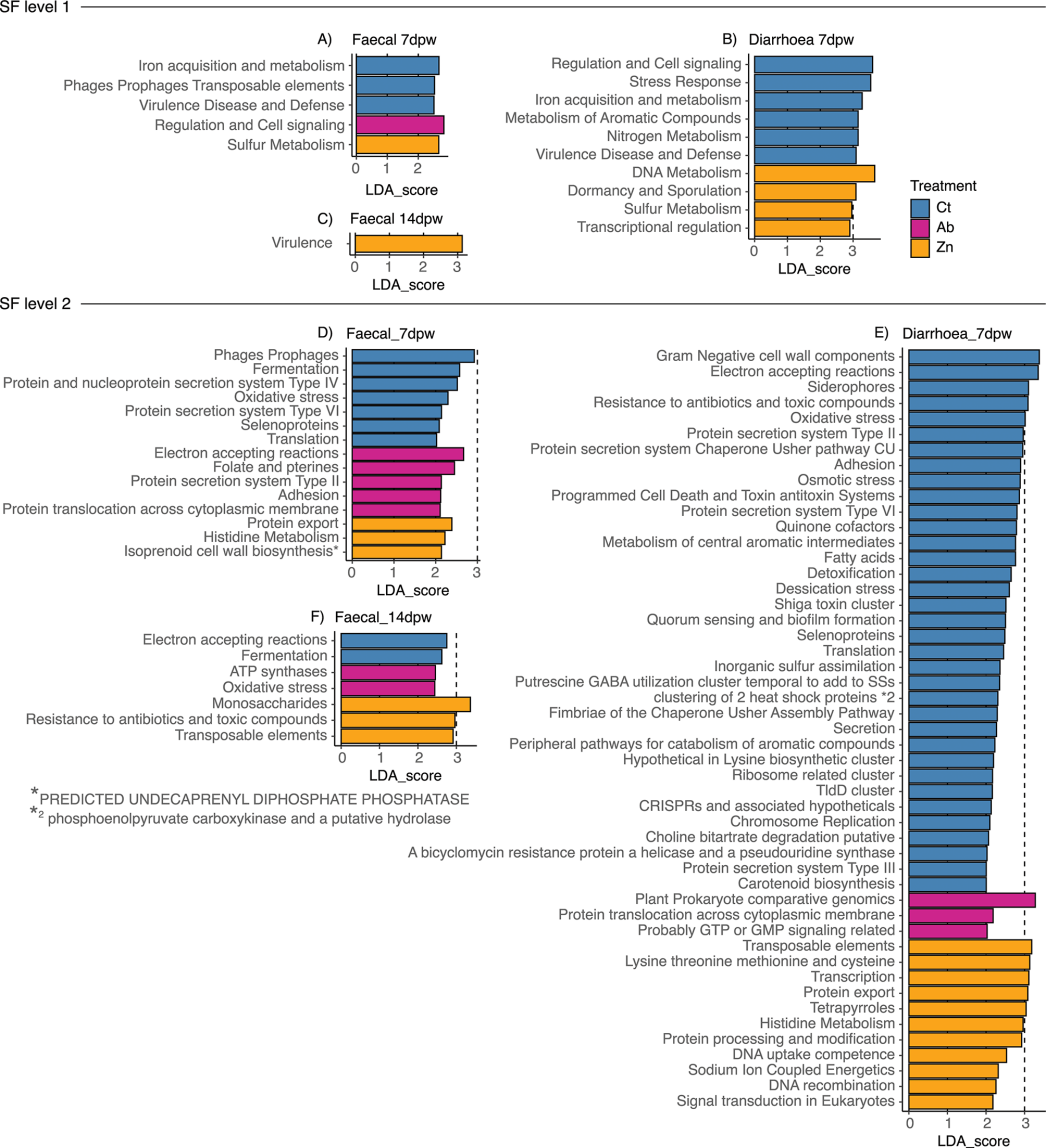
Differences in Super-focus (SF) functional levels 1 (**A**-**C**) and 2 (**D**-**F**), returned by LEfSe. (**A**) Functional SF1 categories associated with each dietary treatment in the analysis of Faecal_7dpw. (**B**) Functional SF1 categories associated with each dietary treatment in the analysis of Diarrhoea_7dpw. (**C**) Functional SF1 categories associated with each dietary treatment in the analysis of Faecal_14dpw. (**D**) Functional SF2 categories associated with each dietary treatment in the analysis of Faecal_7dpw. (**E**) Functional SF2 categories associated with each dietary treatment in the analysis of Diarrhoea_7dpw. (**F**) Functional SF2 categories associated with each dietary treatment in the analysis of Faecal_14dpw. Significant functional categories given by LEfSe (Linear discriminant analysis Effect Size) are coloured according to the treatment to which they are associated to

**Fig. 7:**
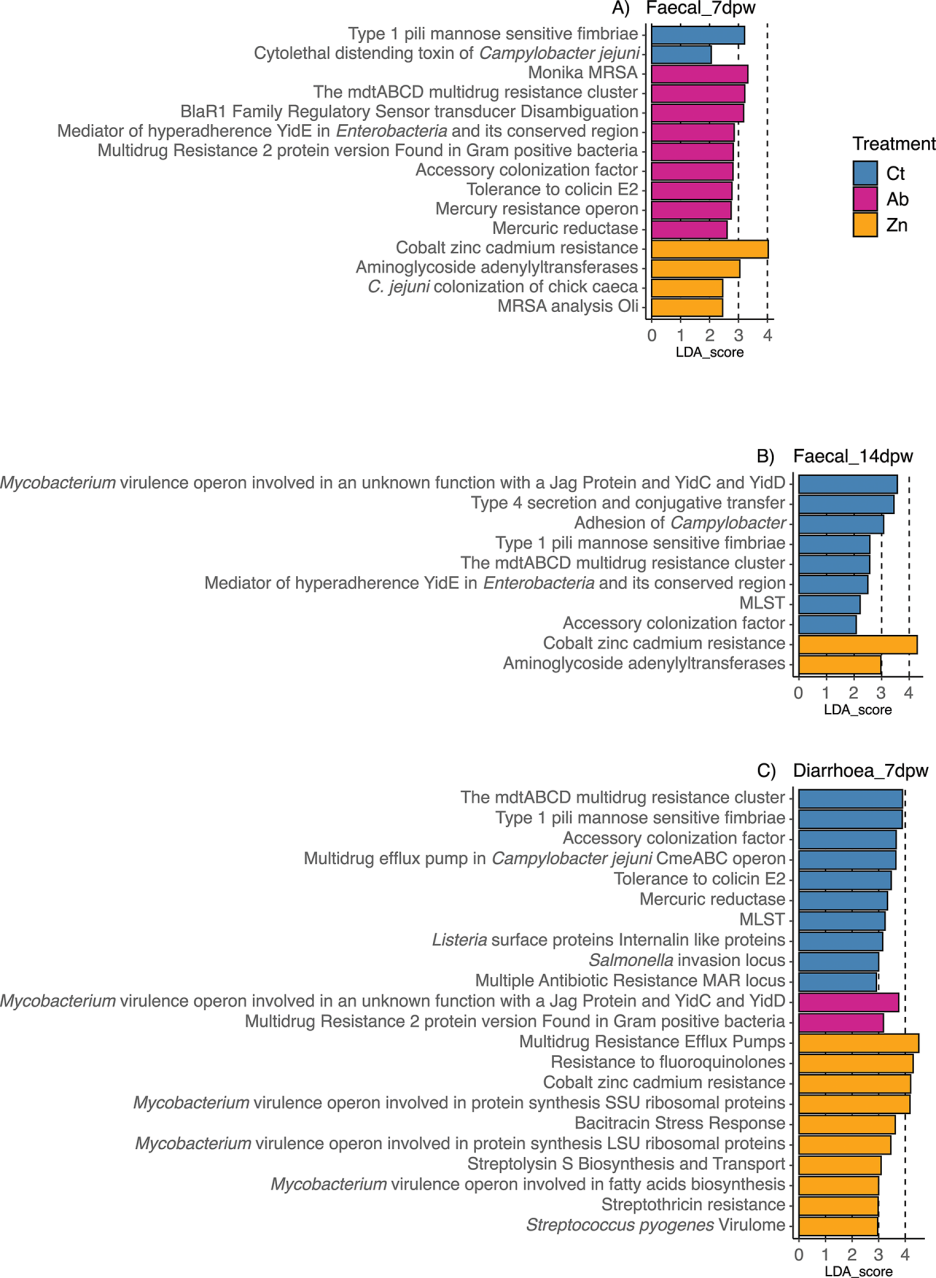
Differences in functions related to Virulence, Disease and Defense of Super-focus (SF) functional level 3. (**A**) Functional Viruelence-related SF3 categories associated with each dietary treatment in the analysis of Faecal_7dpw. (**B**) Functional Virulence-related SF3 categories associated with each dietary treatment in the analysis of Diarrhoea_7dpw. (**C**) Functional Viruelnce-related SF3 categories associated with each dietary treatment in the analysis of Faecal_14dpw. Significant functional categories given by LEfSe (Linear discriminant analysis Effect Size) are coloured according to the treatment to which they are associated to. Functional categories of level 3 with functions related to Virulence, Disease and Defense of SF1

The effect of each treatment on SF1 and SF2 grouped functional categories, as well as SF3 categories related to virulence, disease and defence was investigated separately by consistency-dpw levels (Figs. 6 and 7). LEfSe analyses revealed no associations of any category to any of the treatments at 0dpw. The effects of treatment in SF1 categories at days 7dpw (Faecal and Diarrhoea) and 14dpw are shown in Fig. 6A, B and C, respectively. Among the more interesting broad category functions seen in Faecal_7dpw samples, the categories Phages-Prophages, Protein and nucleoprotein secretion system type IV, Protein secretion system type VI, Oxidative stress were associated to the microbiotas of animals fed the Ct diet, whereas the categories of Protein secretion system Type II and Adhesion were associated to microbiotas from Ab treated pigs. The functions of Protein export, Histidine metabolism, and Isoprenoid cell wall biosynthesis were associated to the Zn group (Fig. 6D). The 14dpw faecal samples from the Ct group were enriched in Electron accepting reactions and Fermentation functions, Ab group in ATP synthases and Oxidative stress while the Zn group exhibited a higher abundance of functions linked to Monosaccharides, Resistance to antibiotics and toxic compounds and Transposable elements (Fig. 6F). Analysis of the Diarrhoea_7dpw samples revealed a high number of categories linked to Virulence in the Ct samples, Protein translocation across cytoplasmatic membrane and GTP or GMP signalling associated with the Ab samples, and functions related to amino acid metabolism (Lysine, threonine, methionine, and cysteine, and histidine metabolism), Protein export, processing and modification, DNA uptake, competence, and recombination, and Transposable elements related to Zn (Fig. 6E).

Analysis of the level 2 subsystem levels revealed the category *Resistance to antibiotics and toxic compounds* associated with the Zn and Ct treatments in Faecal_14dpw and Diarrhoea_7dpw samples, respectively. However, detailed analysis of Virulence, disease, and defence category functions at the lower functional level (SF3) revealed links with more specific antimicrobial resistance mechanisms depending on each consistency-dpw level, and cobalt, zinc and cadmium resistance specifically associated with the Zn treatment (Fig. 7). Functions associated the Ct group were related to mechanisms of virulence of Gram-negative microorganisms, adhesion, and colonization, among others, as well as other mechanisms related to adhesion and multidrug resistance in Ab treated animals (Fig. 7).

### *Escherichia coli* virulence profiling

We further mapped the *E. coli* virulence related proteins and virulence factors using data from PFAM protein families identified using Humann3, and virulence factor genes detected in assembled scaffolds, respectively.

**Fig. 8:**
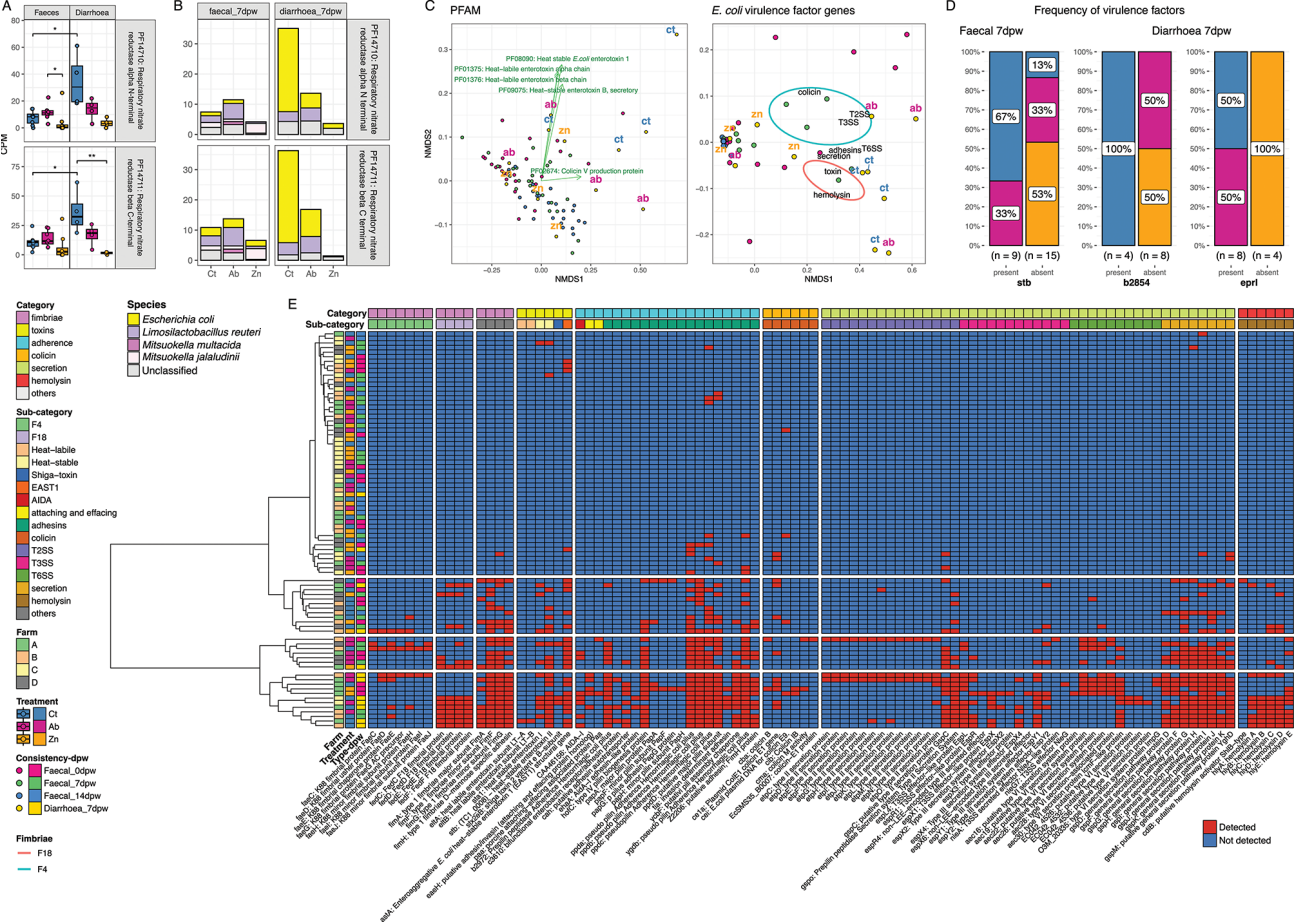
*E. coli* virulence-related metabolic traits and detected virulence factors at 7 days post weaning. (**A**) Abundance of respiratory nitrate reductase subunits PFAMs at 7dpw, in each treatment, separated by consistency. (**B**) Contribution of each species to each nitrate reductase subunit PFAM in each treatment separated by consistency at 7dpw. (**C**) Ordination of samples according to PFAM abundance and virulence factor genes occurrence, using Bray-Curtis and Simple Matching coefficient (SMC), respectively. (**D**) Frequency of significant different virulence factors detected in Faeces and Diarrhoea at 7dpw. (**E**) Heatmap of occurrence of virulence factors in samples clustered by Ward clustering of SMC distances between samples

A higher abundance of both respiratory nitrate reductase subunits was found in Diarrhoea samples from Ct pigs compared to Ct Faecal samples (Fig. 8A). Faecal samples of Ab pigs exhibited a higher abundance of the α N-terminal subunit (PF14710), whereas Ct Diarrhoeic samples had a higher abundance of the β C-terminal subunit (PF14711). The analysis of species associated to these subunits revealed that *E. coli* involvement in non-medicated diarrhoea samples was higher than Zn diarrhoeic samples, as well as greater than all treatments of faecal samples (Fig. 8B).

Ordination of both PFAM and virulence factors genes data using the Bray-Curtis and Simple Matching coefficient, respectively, clearly clustered diarrhoeic samples separately. The effect of treatments was also apparent in the separation of Ct diarrhoeic samples. Heat-labile and Heat-stable toxins influenced the ordination of these samples (envfit, *P* < 0.05), whereas the centroids of the weighted averages of grouped genes according to categories were also located among the Ct and Ab diarrhoeic samples (Fig. 8C). Ct diarrhoeic samples harboured F18 fimbriae (Fig. 8D, supplementary figure S7B). Significant differences were also found in the frequency of the Heat-Stable enterotoxin II (*stb*) in Faecal_7dpw samples, whereas the frequency of the detected *b2854* and *eprI* genes were higher and lower in Ct and Zn diarrhoeic samples, respectively (Fig. 8D). Ward clustering of the SMC distances clearly demonstrated these differences between diarrhoeic samples (bottom branch), and the rest of the samples, in *E. coli* fimbriae, adhesins, toxins, haemolysins and other virulence factors (Fig. 8E).

## Discussion

In-feed ZnO and Ab have been widely used to prevent and control PWD, a disease affecting piglets in one of the most critical moments of the production cycle. Environmental soil pollution and antimicrobial resistance have raised concerns about the regular use of these treatments. Therefore, new approaches to control PWD are needed, and understanding how these products control diarrhoea is crucial to develop new effective strategies to replace them. In this context, the impact of weaning and PWD treatments on the microbiota composition and functionality is gaining attention [8]. Fine tuned characterisation of the microbiota in normal and diarrhoeic faeces from pigs with or without therapeutic treatments will provide knowledge to create a more resilient microbiota at this critical stage, by finding taxonomic and functional traits linked to PWD resistance/onset. Research in this topic is generally carried out on experimental farms, with a single trial or farm per study and the design of which is based on controlled conditions [38, 39]. While this approach helps control the background variability and standardise results, it is also less representative compared to commercial conditions, where higher environmental variability is expected. The microbiota background in a particular farm may determine the effects observed when using Ab and Zn. In this study, we used shotgun metagenomic sequencing to explore the effect of in-feed Ab or ZnO on the piglet microbiota by replicating the same design on different commercial farms, where the three feed strategies (Ab, ZnO, treatment-free (Ct)) were put in place, capturing microbiota common traits and variability among treatments on different farms.

### Post weaning transition

The microbiota richness and diversity increases from weaning onwards, linked to the complex diet and age of the animals [9, 40, 41]. In our study, we observed a shift in microbial dominance towards the first 14dpw (P. evenness, Shannon and Simpson indexes), regardless of the therapeutic treatment used. The lower values observed both for taxa and functions in evenness indexes demonstrate that ZnO medication rapidly establish a hierarchy in the microbiota composition compared to non-medicated or Ab medicated animals. This effect in alpha diversity values was previously reported for taxonomic analyses [17, 42, 43] but here we confirm for the first time its impact on the microbiota functionality as well.

Ordination of samples clearly split samples by dpw both at species and functional level, revealing a quick adaptation and transition to solid feed [9, 40]. Diving into the taxonomical traits driving these differences, Ward clustering of squared Bray Curtis distances split samples into two main branches, defined by the sampling time point and the relative abundance of species. Transition of species from a milk-oriented microbiota (cluster 3) to a cluster dominated by fibre-degrading bacteria (cluster 2) proved the shift in microbiota composition at this stage. Interestingly, cluster 1 included samples from the three sampling time-points, but with lower abundance of milk-oriented bacteria which dominated cluster 3 such as *Clostridiales* bacterium CCNA10 and *Oscillibacter* spp., as well as lower abundance of *Prevotella* spp., *Eubacterium rectale*, and *Roseburia hominis*, in large abundance in cluster 2, and thus associated to 7 and 14dpw samples. These results demonstrate different paces in the intestinal microbiota maturation in piglets after weaning. Finally, all diarrhoea samples clustered together, with an unbalanced microbiota dominated by *E. coli*. The clustering also revealed certain sub-clusters, grouping samples from the same farm or under the same therapeutic treatment, see for instance most of Ab samples from farm D were in cluster 1; Ab samples from farm C in cluster 2; and samples of farm A (four of 0dpw and 1 of diarrhoea) in cluster 4. These sub-clusters revealed the influence of treatments and farms at a lower hierarchical order than dpw and diarrhoea occurrence.

Evolution of the microbial composition post weaning followed a different path depending on the dietary treatment, maintaining some species with a similar relative abundance across days and treatments such as *F. prausnitzii*, *L. reuteri*, *P. ruminicola*, *P. denticola*, *E. rectale*, and some species characteristic of each treatment at each day post weaning. Starting at the day of weaning (0dpw), before any treatments were applied, species relative abundance was virtually the same in all treatments, reflecting the high evenness seen in alpha diversity analysis. The relative abundance patterns of functions grouped in broad categories (super-focus 1) reflected a stable composition throughout the 2 weeks of the study. These results evidence the capability of the microbiota to maintain a necessary pool of functions despite the species succession and abundance fluctuation seen across time-points, and other perturbations like the treatments applied. These concepts are known as resilience and functional redundancy [44, 45].

Regarding the species associated with each dietary treatment, species exhibiting consistency across days post weaning and treatment were those ascribed to the phylum *Pseudomonadota* in Ct animals (*E. coli* in Faecal_7dpw and *D. piger* in 7 and 14dpw– formerly within this Phylum–), and species belonging to family *Bacteroidaceae* (*Bacteroides* spp.) and *Clostridioides difficile* in Faecal_7 and 14dpw in Zn treated animals.

The detrimental effect exerted by ZnO in those bacteria belonging to phylum *Pseudomonadota* (*E. coli*) and other genera such as *Desulfovibrio* spp. (formerly within class *Deltaproteobacteria*, Phylum *Pseudomonadota*) as well as the association of species within *Bacteroidaceae* such as *Bacteroides* spp. and *Parabacteroides* spp. to Zn medication is in accordance with other studies [14, 17, 46]. Indeed, these species, as well as others within this family as *Prevotella* spp. seem to be tolerant to Zn and Ab [14, 17, 47]. We observed a high diversity of *Bacteroides* spp. associated with this treatment. As member of the pig microbiota, *Bacteroides* spp. are Gram negative bacteria associated to lactating animals [9, 40, 48], while in humans, these bacteria are linked to diets with a high content of proteins and fat [49], and several reviewed studies report the high adaptability of these species in the intestinal environment exhibiting cross-feeding abilities, i.e., providing simpler nutrients to the host and other microorganisms within the gut, or modulating the immune response [50]. Studies using in-feed ZnO have also reported anti-inflammatory effects through overexpression of IL-10 mRNA levels [51, 52]. A shotgun sequencing approach enables microbiota characterization at species level. Notably, here we found a highly diverse group of *Bacteroides* spp. associated with Zn treatment. The effects of ZnO on gut physiology might be further driven by species like those within the *Bacteroidaceae* family, exerting a competitive exclusion against pathogenic bacteria and modulating the immune response.

At 7dpw, *Streptococcus suis* and *Akkermansia muciniphila* were also associated with samples from non-medicated (Ct) animals. In this study, ZnO and Ab not only controlled *E. coli* overgrowth, but also *S. suis*, another known pathogen of pigs and a zoonotic agent [53]. *A. muciniphila* is a mucosa-dwelling bacterium which has been associated with mucosa damage in association with *Salmonella* Typhimurium infection [54]. At 14dpw, Ct animals had a higher abundance of *E. coli* as well as *Megasphaera* spp., whereas species associated with diets with antibiotics shifted across days post weaning. For instance, while *M. elsdenii* was linked to the Ab diet in global data, as well as *Dialister massiliensis* and *Selenomonas sputigena* to Ab at 7dpw, these species were associated with Ct animals at 14dpw. *M. elsdenii* is a member of the pig’s microbiota, which increases in abundance in the microbial community as the animals consume feed [7], and which has been reported to rise in abundance in animals fed Ab containing diets [17, 55, 56]. Species from the genera *Megasphaera* and *Diallister* spp., all from the *Veillonellaceae* family, exhibited a higher abundance in Ct pigs 14dpw, and decreased in abundance in Zn treated animals on all dpw. Some of these mentioned members appear to be susceptible to ZnO or the conditions caused by it in the gut [14, 17, 57].

### The microbiota composition in diarrhoeal samples and the role of Ab and Zn lessening its alterations in the first two weeks post weaning

Several studies have assessed the effect of certain antibiotics, therapeutic concentrations of ZnO, and ZnO alternative products such as Zn nanoparticles or other alternative feedstuff products, on the development of the microbiota in pigs and reported minor differences between different zinc alternative treatments, being not as effective as pharmaceutical doses of ZnO in preventing weaning associated alterations and PWD [58]. In this study, we moved a step forward exploring the effects of both treatments on the microbiota composition and functionality in clinical diarrhoea samples, finding large differences between treatments.

Firstly, ordination of the samples revealed a remarkable difference between diarrhoea samples and all the other groups. Interestingly, 3 of 4 diarrhoea samples from the Ct group were located far apart from the rest of the samples, whereas 3 of 4 samples of Ab group were located closer to non-diarrhoeic faeces and samples from ZnO treated animals but with diarrhoeic consistency seemed to be scattered within the non-diarrhoeic faeces. Indeed, representation of Ward Clustering of Bray Curtis distances onto NMDS ordination space revealed more complex relationships between these samples; four diarrhoea samples (3 from control pigs and another from Ab medicated pigs) clustered into an isolated group while the rest of diarrhoea samples were scattered into other clusters of the dendrogram. These relationships were similar at afunctional level (Fig. 2C), where Ab and Zn diarrhoea samples were located closer to healthy animals. These results might suggest that the Ab and especially Zn, revert the microbiota composition and functionality to its normal state. Indeed, the microbiota transition in the period of two weeks post weaning was disrupted in control pigs and to a lesser extent in Ab treated pigs. Diarrhoeic samples from these two groups exhibited an increase of *E. coli* (in Ct 24.95% and Ab 8.46%) and *M. elsdenii* in Ab treated animals (8.65%) (Fig. 4A), whereas the microbiota profile in diarrhoeic samples of Zn treated pigs resembled Faecal_7dpw samples. Both Ab and Zn prevented *E. coli* overgrowth in healthy and diarrhoeic samples at 7dpw. This finding might explain why, in the absence of Ab and Zn, PWD can result in an imbalance dominated by *E. coli*, with large increases in loss of performance and mortality.

Detailed analyses of SF1 functional categories among the three groups in diarrhoea samples revealed that slight differences in the Ct broad community functions exerted a great influence on species composition and host homeostasis, thus describing the differences behind the clinical sign of diarrhoea. A higher abundance of functions such as Iron acquisition and metabolism, Prophages, transposable elements, Virulence, disease and defence may indicate signs of an unstable microbial state in the Faecal_7dpw and Diarrhoea_7dpw samples in Ct animals. Sulphur metabolism was enriched in pigs medicated with Zn in Faecal_7dpw and Diarrhoea_7dpw samples, as well as DNA metabolism, transcriptional regulation, and Dormancy and Sporulation in Diarrhoea_7dpw. A higher abundance of this last category may suggest bacterial adaptation to the unfavourable conditions generated either by the state of intestinal dysbiosis, the ZnO effects on the gut, or both. Zn animal samples at 14dpw were linked with functions associated with metals (zinc, cobalt and cadmium resistance) and antimicrobial resistance. At the higher level of functional hierarchy, functions associated with Ct pigs, particularly in diarrhoea samples, were related to adhesion and invasion. Previous studies performed in vitro reported a reduced bacterial adhesion and invasion of ETEC in human Caco-2 cells, and a mild inhibition of biofilm formation by *E. coli* when using zinc oxide or zinc [59, 60]. Similar results were obtained in a previous study in animals receiving a Ct diet at the 7th day post weaning [17].

Taxonomic and functional results were further explained by the *E. coli* virulence profiling. A higher abundance of respiratory nitrate reductase abundance was found in Ct diarrhoeic samples (Fig. 8A and B) which may indicate growth advantage mechanisms being deployed by a potential pathogenic *E. coli* in an inflamed intestine [8, 61, 62]. To the authors knowledge, this is the first study describing results regarding this competitive mechanism, using metagenomics, in pigs PWD. The results also evidenced ZnO as the best compound controlling potentially pathogenic *E. coli* overgrowth or even presence (Fig. 8C and E), whereas the antibiotic only reduced its abundance, harbouring nearly the same virulence factors as detected in Ct samples.

The results found in this study show signs of the potential of *E. coli* as a causative agent of a dysbiosis state within the gut, which are prevented or reverted by ZnO and Ab treatments. Future studies focused on microbiota activity such as meta-transcriptomics or meta-proteomics will help to confirm these findings.

## Conclusions

Overall, the results demonstrated how the intestinal piglet microbiota adapts to weaning, shifting rapidly within the first two weeks after weaning, and that diarrhoea markedly modifies this adaptation. In feed medication, either with antibiotics or Zn, also modulates the transition of species and functional richness, diversity and composition of the microbiota observed in this post-weaning period. Both therapeutic strategies inhibit *E. coli* overgrowth. In addition, while the use of Ab favours the colonisation by *Veillonelaceae* family the use of ZnO seems to exert the contrary effect, but the latter treatment gives advantage to other species within the *Tannerellaceae* and *Bacteroidaceae* familiae. This effect of Ab and Zn use is much more evident when looking at animals with clinical diarrhoea, a result not described in such detail to date. These results map the microbiota of piglets at weaning and may be useful to find alternatives to antimicrobial compounds to keep or restore the microbial equilibrium at the most critical days of the post-weaning period.

### Electronic supplementary material

Below is the link to the electronic supplementary material.

Supplementary Material 1

Supplementary Material 2

Supplementary Material 3

Supplementary Material 4

Supplementary Material 5

Supplementary Material 6

Supplementary Material 7

Supplementary Material 8

Supplementary Material 9

Supplementary Material 10

Supplementary Material 11

Supplementary Material 12

## Data Availability

The sequences from all samples, including negative and positive controls, as well as its associated metadata have been submitted to NCBI Sequence Read Archive (SRA) and will be available under Bioproject accession PRJNA984595: https://dataview.ncbi.nlm.nih.gov/object/PRJNA984595?reviewer=c8elikvj2ftna8sm15k2e9tuk.

## Abbreviations

Ab: Antibiotic
Ct: Control
Dpw: Day post weaning
ETEC: Enterotoxigenic *Escherichia coli*
LDA: Linear Discriminant Analysis
LEfSe: Linear Discriminant Analysis Effect Size
PWD: Post Weaning Diarrhoea
SF: Super-Focus
SF1: Super-Focus level 1
SF2: Super-Focus level 2
SF3: Super-Focus level 3
SMC: Simple Matching Coefficient
Zn: Zinc
ZnO: Zinc Oxide
