# Supplementary figures and images for: Effects of removing in-feed antibiotics and zinc oxide on the taxonomy and functionality of the microbiota in post weaning pigs
Source: Anim Microbiome. 2024 Apr 16;6:18. doi: 10.1186/s42523-024-00306-7 (PMC11022352; doi:10.1186/s42523-024-00306-7)

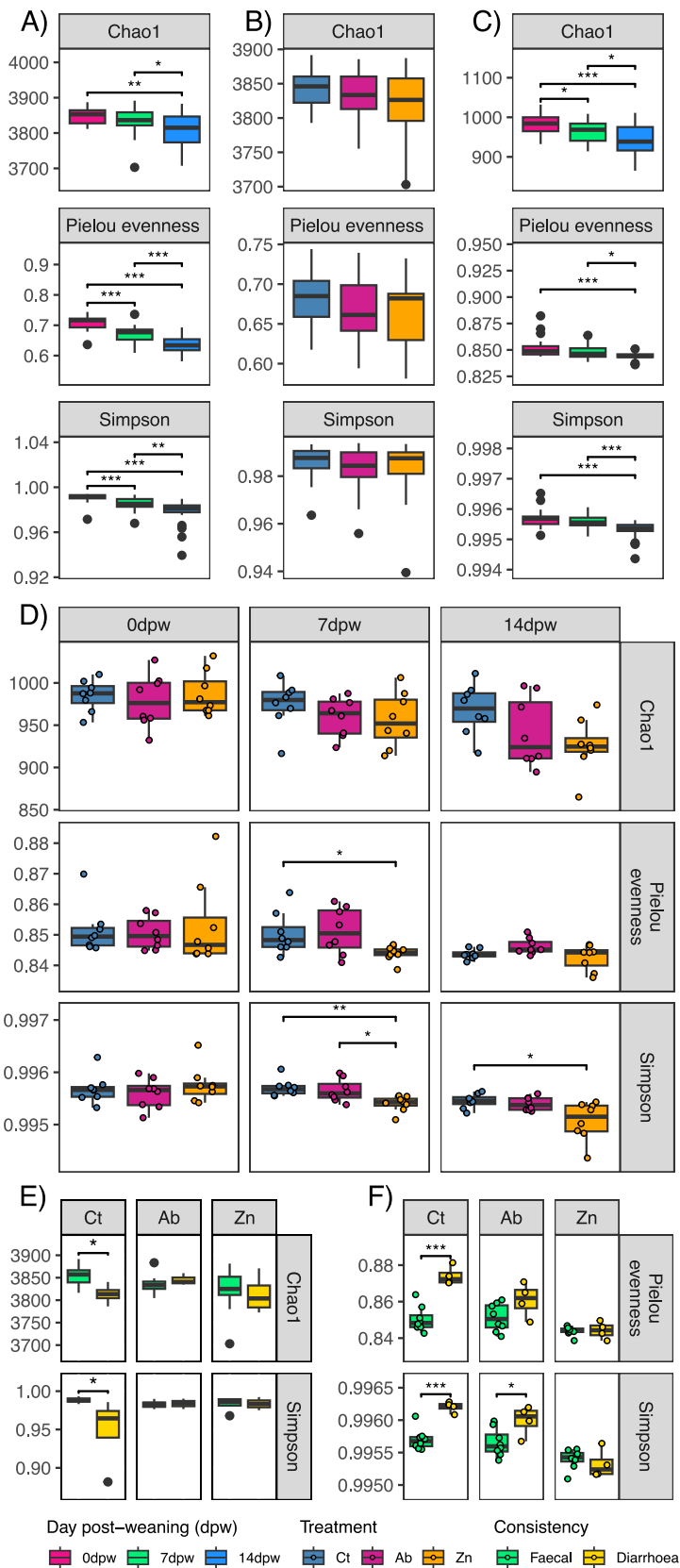

Supplement: Supplementary file 1 — Supplementary Material 1 [file 42523_2024_306_MOESM1_ESM.pdf]

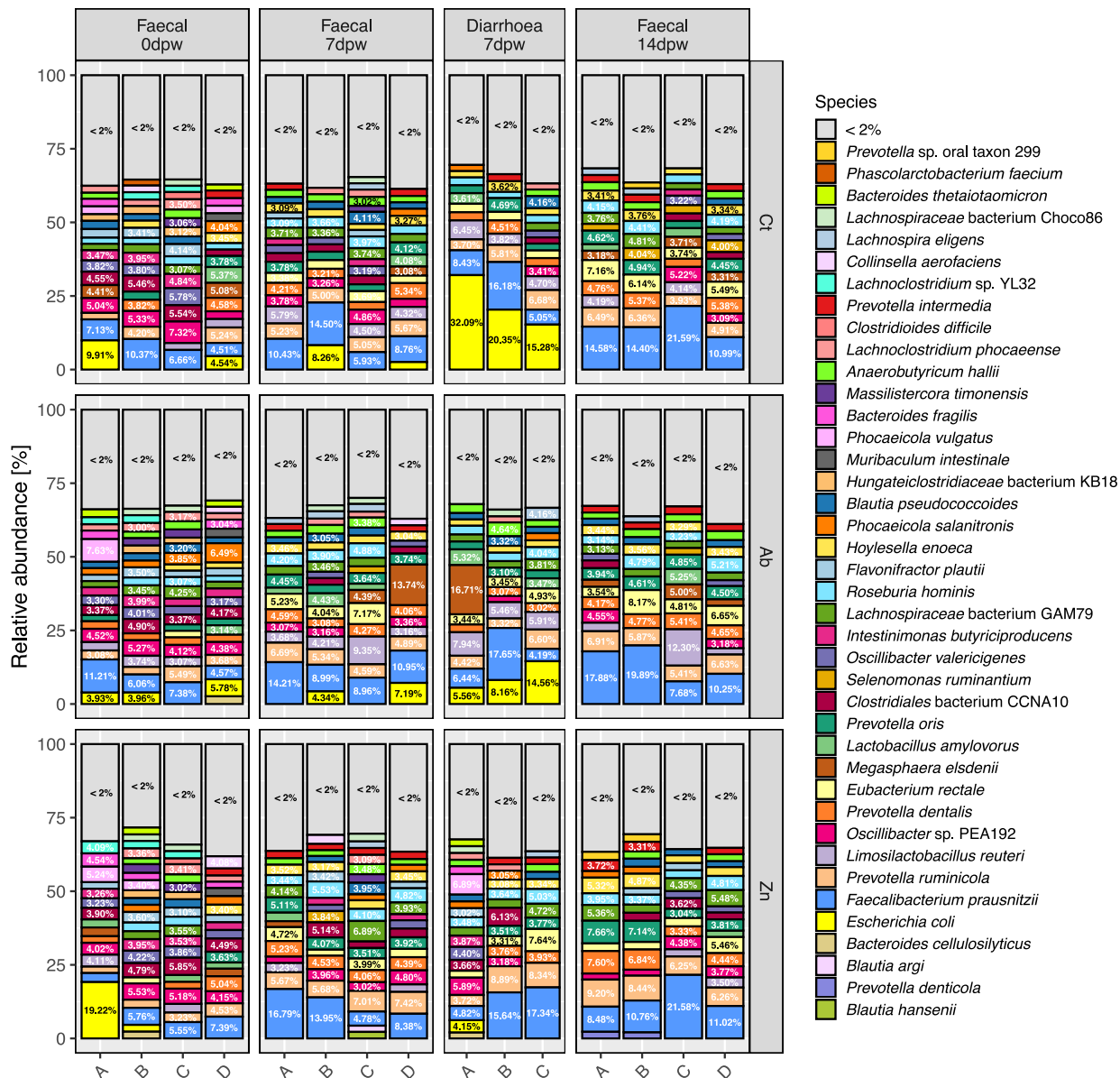

Supplement: Supplementary file 2 — Supplementary Material 2 [file 42523_2024_306_MOESM2_ESM.pdf]

Diarrhoea\_7dpw

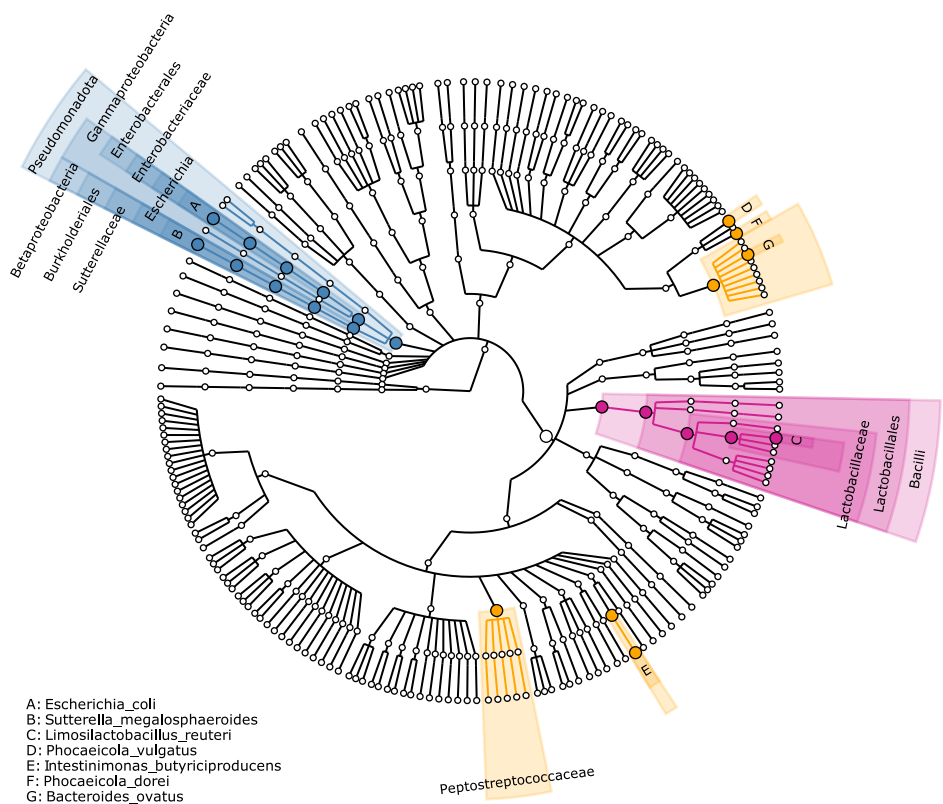

Supplement: Supplementary file 5 — Supplementary Material 5 [file 42523_2024_306_MOESM5_ESM.pdf]

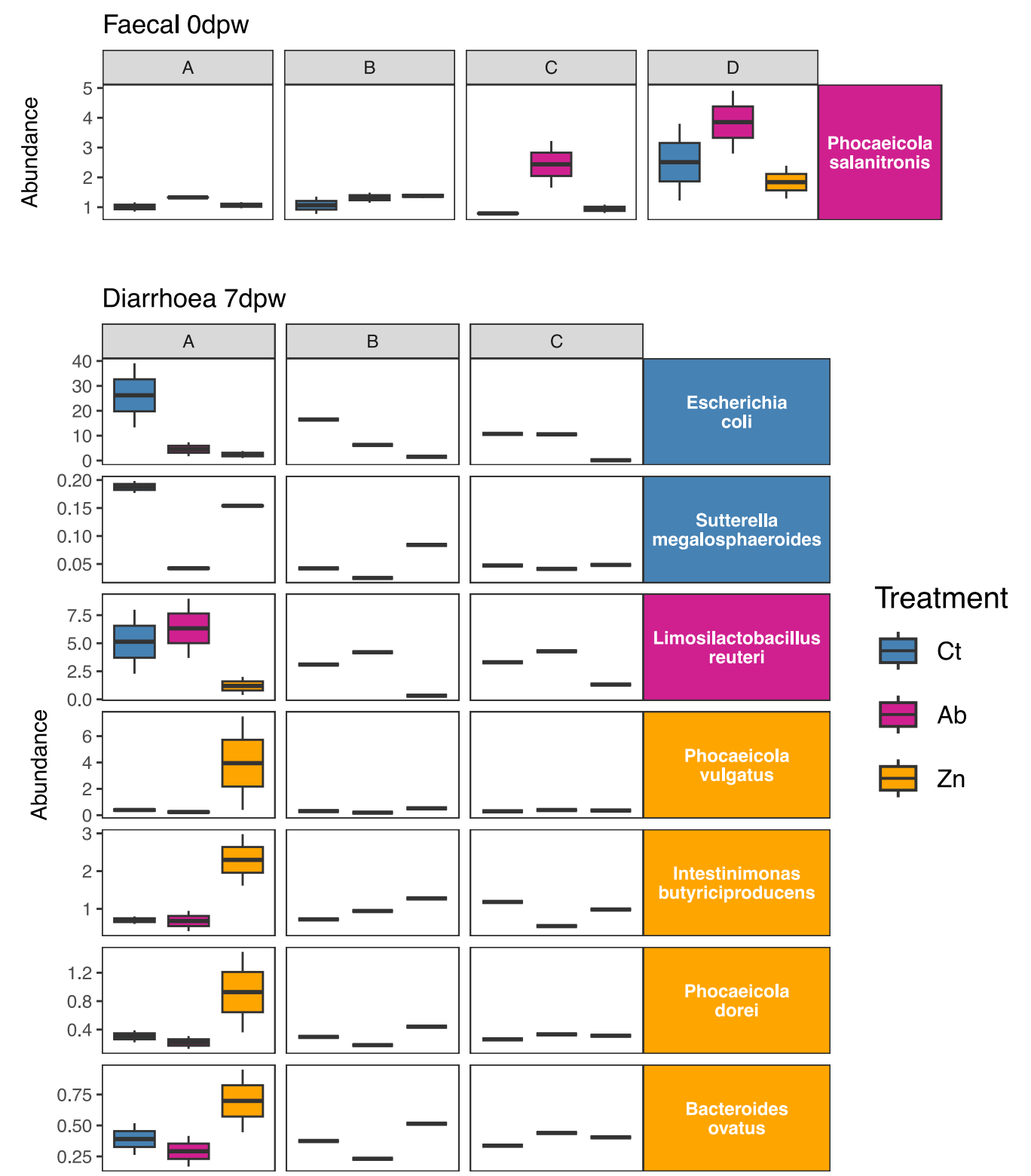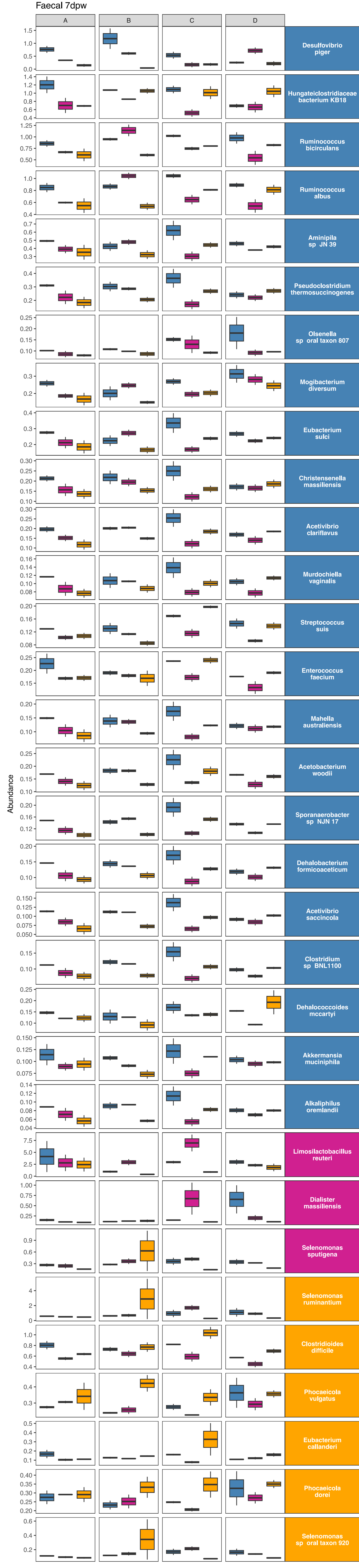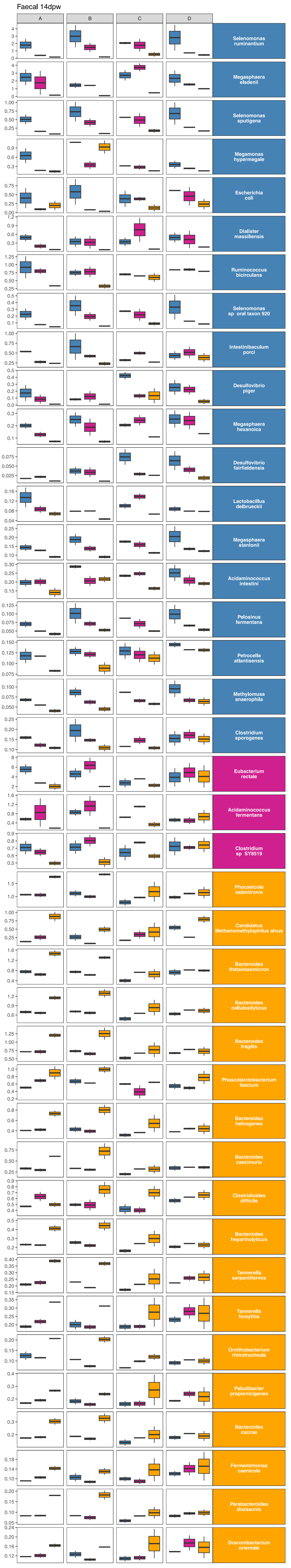

Supplement: Supplementary file 6 — Supplementary Material 6 [file 42523_2024_306_MOESM6_ESM.pdf]

Consistency\_dpw

A Faecal\_7dpw Diarrhoea\_7dpw

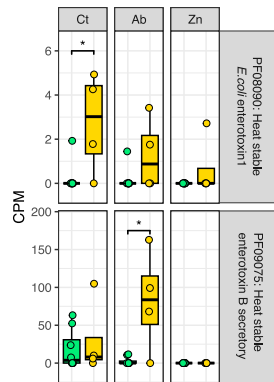

B

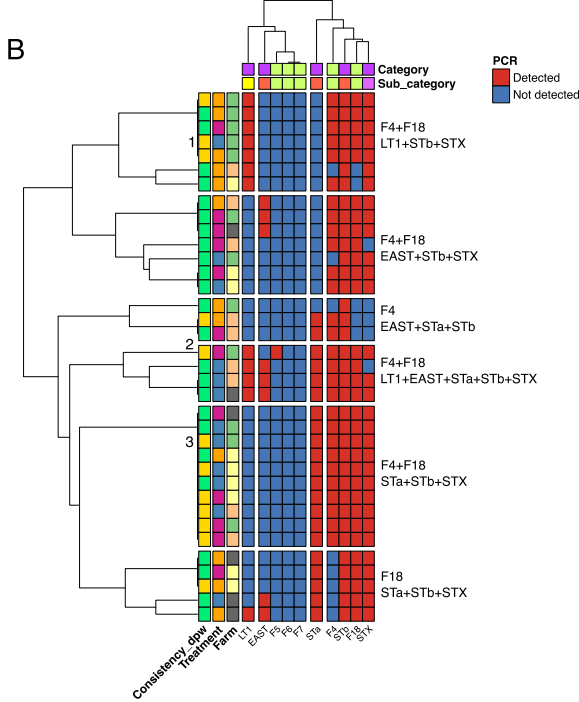

Supplement: Supplementary file 7 — Supplementary Material 7 [file 42523_2024_306_MOESM7_ESM.pdf]
