# Supplementary material for: Effects of removing in-feed antibiotics and zinc oxide on the taxonomy and functionality of the microbiota in post weaning pigs
Source: Anim Microbiome. 2024 Apr 16;6:18. doi: 10.1186/s42523-024-00306-7 (PMC11022352; doi:10.1186/s42523-024-00306-7)
Supplement: Supplementary file 4 — Supplementary Material 4 [file 42523_2024_306_MOESM4_ESM.pdf]

Faecal 14dpw

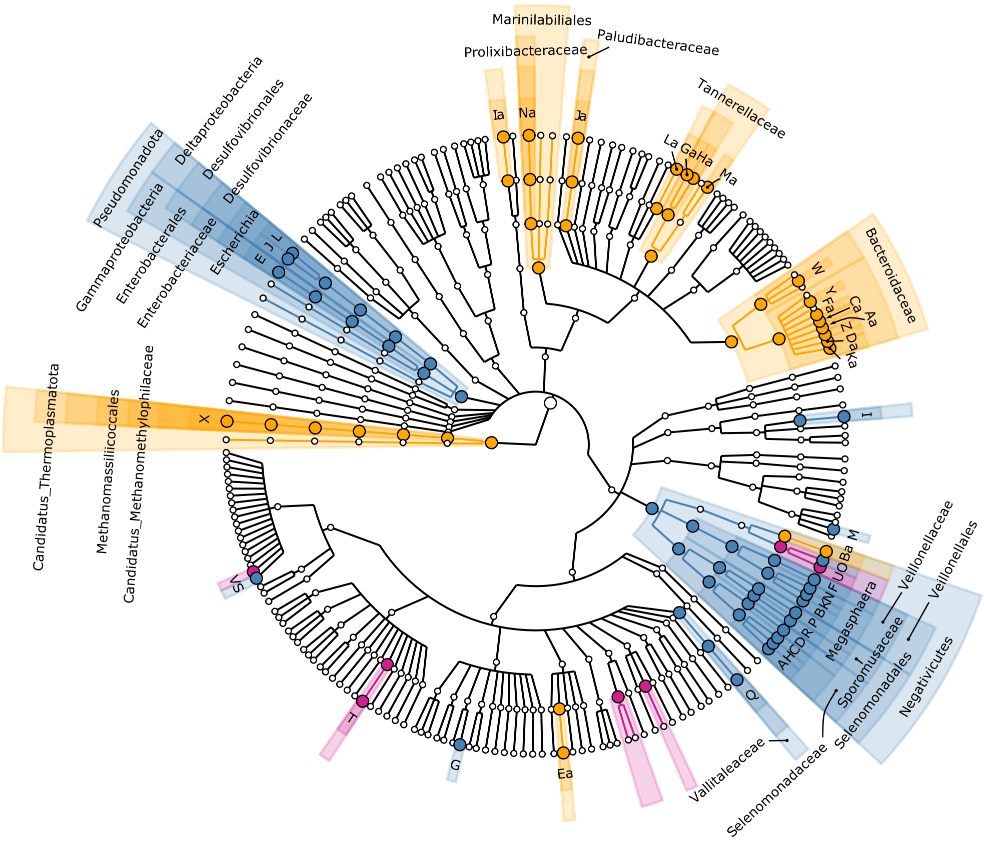

A: Selenomonas_ruminantium
Aa: Bacteroides_fragilis
B: Megasphaera_elsdenii
Ba: Phascolarctobacterium_faecium
C: Selenomonas_sputigena
Ca: Bacteroides_helcogenes
D: Megamonas_hypermegale
Da: Bacteroides_caecimuris
E: Escherichia_coli
Ea: Clostridioides_difficile
F: Dialister_massiliensis
Fa: Bacteroides_heparinolyticus
G: Ruminococcus_bicirculans
Ga: Tannerella_serpentiformis
H: Selenomonas_sp__oral_taxon_920
Ha: Tannerella_forsythia
I: Intestinibaculum_porci
Ia: Ornithobacterium_rhinotracheale
J: Desulfovibrio_piger
Ja: Paludibacter_propionicigenes
K: Megasphaera_hexanoica
Ka: Bacteroides_caccae
L: Desulfovibrio_fairfieldensis
La: Fermentimonas_caenicola
M: Lactobacillus_delbrueckii
Ma: Parabacteroides_distasonis
N: Megasphaera_stantonii
Na: Draconibacterium_orientale
O: Acidaminococcus_intestini
P: Pelosinus_fermentans
Q: Petrocella_atlantisensis
R: Methylomusa_anaerophila
S: Clostridium_sporogenes
T: Eubacterium_rectale
U: Acidaminococcus_fermentans
V: Clostridium_sp__SY8519
W: Phocaeicola_salanitronis
X: Candidatus_Methanomethylophilus_alvus
Y: Bacteroides_thetaiotaomicron
Z: Bacteroides_cellulosilyticus
