# Supplementary material for: Effects of removing in-feed antibiotics and zinc oxide on the taxonomy and functionality of the microbiota in post weaning pigs
Source: Anim Microbiome. 2024 Apr 16;6:18. doi: 10.1186/s42523-024-00306-7 (PMC11022352; doi:10.1186/s42523-024-00306-7)
Supplement: Supplementary file 8 — Supplementary Material 8 [file 42523_2024_306_MOESM8_ESM.pdf]

Supplementary table S1. P-value(R^2) results returned by envfit() function of vegan R package.

Factor	Global	Faecal 0dpw	Faecal 7dpw	Faecal 14dpw
**Species**				
Treatment	0.139(0.041)	0.948(0.017)	0.352(0.098)	0.057(0.195)
Dpw	0.001***(0.407)			
Treat:dpw	0.001***(0.484)			
**Functional**				
Treatment	0.329(0.028)	0.924(0.021)	0.054(0.193)	0.146(0.148)
Dpw	0.001***(0.252)			
Treat:dpw	0.001***(0.341)			
