# Supplementary material for: Effects of removing in-feed antibiotics and zinc oxide on the taxonomy and functionality of the microbiota in post weaning pigs
Source: Anim Microbiome. 2024 Apr 16;6:18. doi: 10.1186/s42523-024-00306-7 (PMC11022352; doi:10.1186/s42523-024-00306-7)
Supplement: Supplementary file 9 — Supplementary Material 9 [file 42523_2024_306_MOESM9_ESM.pdf]

Supplementary Table S2. PERMANOVA results on Global data. P. values are indicated for each factor and level. R2 values are indicated within parenthesis.

Factor	Species	Functional
Treat	0.042*(0.038)	0.121(0.031)
Ct vs Ab	0.62(0.015)	0.677(0.014)
Ct vs Zn	0.24(0.039)	0.384(0.036)
Zn vs Ab	0.24(0.031)	0.646(0.019)
Dpw	P < 0.001***(0.326)	P < 0.001***(0.294)
0dpw vs 7dpw	0.002**(0.250)	0.002**(0.208)
0dpw vs 14dpw	0.002**(0.384)	0.002**(0.336)
7dpw vs 14dpw	0.002**(0.083)	0.002**(0.094)
treat:dpw	0.282(0.043)	0.527 (0.038)
