# Supplementary material for: Effects of removing in-feed antibiotics and zinc oxide on the taxonomy and functionality of the microbiota in post weaning pigs
Source: Anim Microbiome. 2024 Apr 16;6:18. doi: 10.1186/s42523-024-00306-7 (PMC11022352; doi:10.1186/s42523-024-00306-7)
Supplement: Supplementary file 10 — Supplementary Material 10 [file 42523_2024_306_MOESM10_ESM.pdf]

Supplementary table S3. P.value(R^2) results of PERMANOVA analysis performed within each consistency-dpw level.

Factor	0dpw	7dpw	14dpw
**Species**			
Treat	0.961(0.043)	0.042*(0.142)	0.008**(0.193)
Ct vs Ab	0.995(0.045)	0.205(0.096)	0.561(0.053)
Ct vs Zn	0.995(0.014)	0.084(0.132)	0.022*(0.205)
Zn vs Ab	0.995(0.043)	0.173(0.104)	0.022*(0.170)
**Functional**			
Treat	0.972(0.034)	0.022*(0.152)	0.047*(0.161)
Ct vs Ab	0.958(0.029)	0.407(0.066)	0.589(0.052)
Ct vs Zn	0.958(0.020)	0.012*(0.166)	0.132(0.172)
Zn vs Ab	0.958(0.030)	0.104(0.121)	0.132(0.126)
