# Supplementary material for: Effects of removing in-feed antibiotics and zinc oxide on the taxonomy and functionality of the microbiota in post weaning pigs
Source: Anim Microbiome. 2024 Apr 16;6:18. doi: 10.1186/s42523-024-00306-7 (PMC11022352; doi:10.1186/s42523-024-00306-7)
Supplement: Supplementary file 11 — Supplementary Material 11 [file 42523_2024_306_MOESM11_ESM.pdf]

Supplementary table S4. Table of results of PERMANOVA and envfit analysis performed in Diarrhoea samples data.

PERMANOVA		
Factor	Species	Functional
Treat	0.035*(0.295)	0.004**(0.447)
Ct vs Zn	0.087.(0.316)	0.076.(0.478)
Ct vs Ab	0.297 (0.159)	0.076.(0.314)
Zn vs Ab	0.087.(0.236)	0.076.(0.263)
envfit	Species	Functional
Treat	0.108(0.339)	0.0047**(0.508)
