# Supplementary material for: Effects of removing in-feed antibiotics and zinc oxide on the taxonomy and functionality of the microbiota in post weaning pigs
Source: Anim Microbiome. 2024 Apr 16;6:18. doi: 10.1186/s42523-024-00306-7 (PMC11022352; doi:10.1186/s42523-024-00306-7)
Supplement: Supplementary file 12 — Supplementary Material 12 [file 42523_2024_306_MOESM12_ESM.docx]

## Supplementary information

**Additional file 1: Supplementary figure S1. Analysis of microbiota α-diversity indexes Chao1, Pielou evenness and Simpson at species and functional levels, by day post weaning (dpw) and treatment.** A) Results of α-diversity at species level, by day post-weaning (dpw). B) Alpha diversity values by treatment factor, at species level. C) Results of α-diversity by dpw, at functional level (Super-focus level 3 category). D) Results of α-diversity analysis by treatment within each day post weaning, performed at functional level (Super-focus level 3 category). E) Results of α-diversity analysis in 7dpw samples, performed at species level, comparing normal and diarrhoeic faeces and. F) Results of α-diversity analysis in 7dpw samples, performed at functional level (Super-focus level 3 category), comparing normal and diarrhoeic faeces in each dietary treatment group. *P < 0.05, **P < 0.01, and ***P < 0.001. Sequences were taxonomically and functionally assigned using Kaiju and Super-focus, respectively.

**Additional file 2: Supplementary figure S2.** Comparison of mean relative abundance of the most representative species at each farm in each consistency-dpw group, separated by treatments.

**Additional file 3: Supplementary figure S3.** A) Species associated with each dietary treatment in the analysis of Faecal_0dpw species data, according to LEfSe (Linear discriminant analysis Effect Size), most likely explaining the differences among dietary treatments. B) Taxa associated with each dietary treatment in the analysis of Faecal_7dpw data. Significant species are coloured according to the treatment to which they are associated to, and are annotated in the cladogram as letters, which can be identified below it.

**Additional file 4: Supplementary figure S4.** Taxa associated with each dietary treatment in the analysis of Faecal_14dpw data. Significant species are coloured according to the treatment to which they are associated to, and are annotated in the cladogram as letters, which can be identified below it.

**Additional file 5: Supplementary figure S5.** Taxa associated with each dietary treatment in the analysis of Diarrhoea_7dpw data. Significant species are coloured according to the treatment to which they are associated to, and are annotated in the cladogram as letters, which can be identified below it.

**Additional file 6: Supplementary figure S6:** Relative abundance of the species explaining the differences among treatments in each consistency-dpw level, according to LEfSe analysis. The data is split by farms to compare the effect of the treatments in each one of the significant species at each farm.

**Additional file 7: Supplementary figure S7.** Comparison of PFAM associated to ETEC toxins between Faecal_7dpw and Diarrhoea_7dpw, separated by treatments. A) Abundance of *E. coli* Heat Stable enterotoxins between faecal and diarrhoea samples at 7dpw, separated by treatments. B) Toxins and fimbriae present in samples detected by PCR. C) Comparison of *E. coli* toxins abundance amongst treatments at 7dpw.

**Additional file 8: Supplementary table S1.** P-value(R^2^) results returned by envfit() function of vegan R package.

**Additional file 9: Supplementary Table S2.** PERMANOVA results on Global data. P. values are indicated for each factor and level. R^2^ values are indicated within parenthesis.

**Additional file 10: Supplementary table S3.** P.value(R^2^) results of PERMANOVA analysis performed within each consistency-dpw level.

**Additional file 11: Supplementary table S4.** Table of results of PERMANOVA and envfit analysis performed in Diarrhoea samples data.
