# Supplementary material for: Effects of removing in-feed antibiotics and zinc oxide on the taxonomy and functionality of the microbiota in post weaning pigs
Source: Anim Microbiome. 2024 Apr 16;6:18. doi: 10.1186/s42523-024-00306-7 (PMC11022352; doi:10.1186/s42523-024-00306-7)
Supplement: Supplementary file 3 — Supplementary Material 3 [file 42523_2024_306_MOESM3_ESM.pdf]

A)

Faecal 0dpw

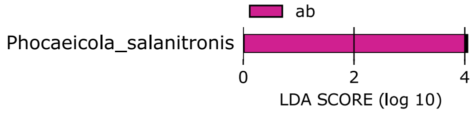

B)

Faecal_7dpw

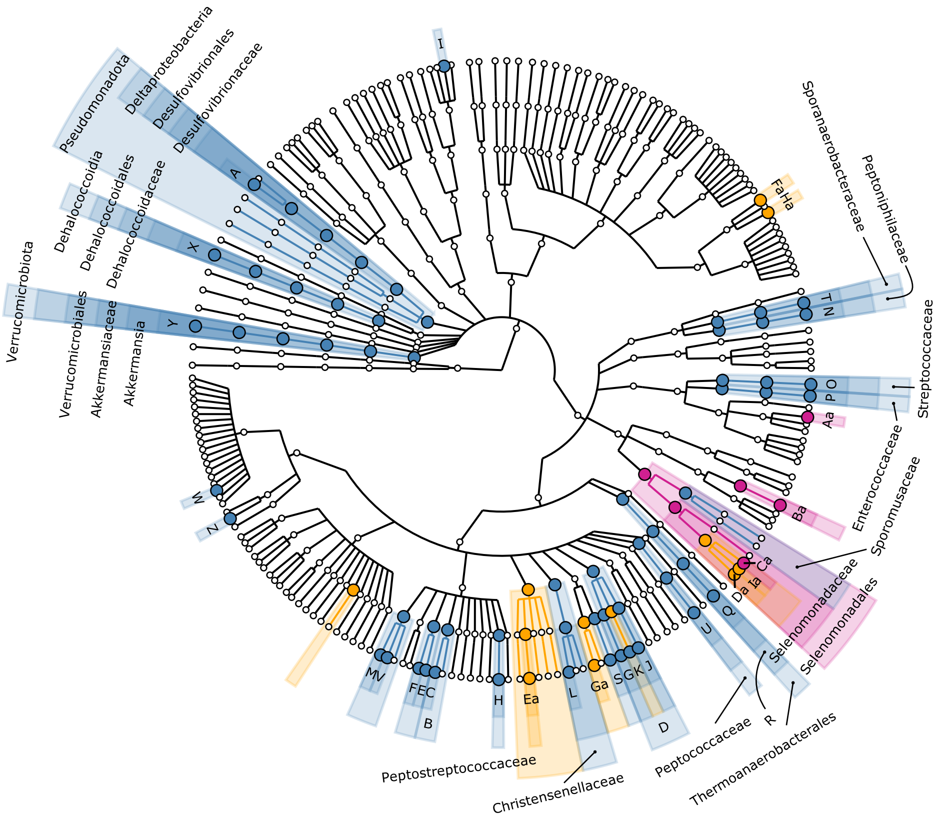

A: Desulfovibrio_piger
Aa: Limosilactobacillus_reuteri
B: unclassified_Oscillospiraceae
Ba: Dialister_massiliensis
C: Hungateiclostridiaceae_bacterium_KB18
Ca: Selenomonas_sputigena
D: Eubacteriales_Family_XIII__Incertae_Sedis
Da: Selenomonas_ruminantium
E: Ruminococcus_bicirculans
Ea: Clostridioides_difficile
F: Ruminococcus_albus
Fa: Phocaeicola_vulgatus
G: Aminipila_sp__JN_39
Ga: Eubacterium_callanderi
H: Pseudoclostridium_thermosuccinogenes
Ha: Phocaeicola_dorei
I: Olsenella_sp__oral_taxon_807
Ia: Selenomonas_sp__oral_taxon_920
J: Mogibacterium_diversum
K: Eubacterium_sulci
L: Christensenella_massiliensis
M: Acetivibrio_clariflavus
N: Murdochiella_vaginalis
O: Streptococcus_suis
P: Enterococcus_faecium
Q: Mahella_australiensis
R: Thermoanaerobacterales_Family_IV__Incertae_Sedis
S: Acetobacterium_woodii
T: Sporanaerobacter_sp__NJN_17
U: Dehalobacterium_formicoaceticum
V: Acetivibrio_saccincola
W: Clostridium_sp__BNL1100
X: Dehalococcoides_mccartyi
Y: Akkermansia_muciniphila
Z: Alkaliphilus_oremlandii
